# Complexome profiling on the *Chlamydomonas lpa2* mutant reveals insights into PSII biogenesis and new PSII associated proteins

**DOI:** 10.1093/jxb/erab390

**Published:** 2021-08-26

**Authors:** Benjamin Spaniol, Julia Lang, Benedikt Venn, Lara Schake, Frederik Sommer, Matthieu Mustas, Stefan Geimer, Francis-André Wollman, Yves Choquet, Timo Mühlhaus, Michael Schroda

**Affiliations:** 1 Molekulare Biotechnologie & Systembiologie, TU Kaiserslautern, Paul-Ehrlich Straße 23, D-67663 Kaiserslautern, Germany; 2 Computational Systems Biology, TU Kaiserslautern, Paul-Ehrlich Straße 23, D-67663 Kaiserslautern, Germany; 3 Biologie du Chloroplaste et Perception de la Lumière chez les Microalgues, Institut de Biologie Physico-Chimique, UMR CNRS/UPMC 7141, Paris, France; 4 Zellbiologie/Elektronenmikroskopie, Universität Bayreuth, 95440 Bayreuth, Germany; 5 University of Cambridge, UK

**Keywords:** BN-PAGE, *Chlamydomonas*, comigration profiles, complexome profiling*s*, CP43, mass spectrometry, photosystem I antenna, photosystem II biogenesis, photosystem II subunits, thylakoid membranes

## Abstract

While the composition and function of the major thylakoid membrane complexes are well understood, comparatively little is known about their biogenesis. The goal of this work was to shed more light on the role of auxiliary factors in the biogenesis of photosystem II (PSII). Here we have identified the homolog of LOW PSII ACCUMULATION 2 (LPA2) in *Chlamydomonas*. A *Chlamydomonas reinhardtii lpa2* mutant grew slower in low light, was hypersensitive to high light, and exhibited aberrant structures in thylakoid membrane stacks. Chlorophyll fluorescence (*F*_v_/*F*_m_) was reduced by 38%. Synthesis and stability of newly made PSII core subunits D1, D2, CP43, and CP47 were not impaired. However, complexome profiling revealed that in the mutant CP43 was reduced to ~23% and D1, D2, and CP47 to ~30% of wild type levels. Levels of PSI and the cytochrome *b*_6_*f* complex were unchanged, while levels of the ATP synthase were increased by ~29%. PSII supercomplexes, dimers, and monomers were reduced to ~7%, ~26%, and ~60% of wild type levels, while RC47 was increased ~6-fold and LHCII by ~27%. We propose that LPA2 catalyses a step during PSII assembly without which PSII monomers and further assemblies become unstable and prone to degradation. The LHCI antenna was more disconnected from PSI in the *lpa2* mutant, presumably as an adaptive response to reduce excitation of PSI. From the co-migration profiles of 1734 membrane-associated proteins, we identified three novel putative PSII associated proteins with potential roles in regulating PSII complex dynamics, assembly, and chlorophyll breakdown.

## Teaser

We identify three novel proteins potentially associated with PSII and a role for auxiliary factor LPA2 in catalysing a step required for stable PSII assembly beyond PSII monomers in *Chlamydomonas*.

## Introduction

Photosystem (PS) II is a light-driven water:plastoquinone oxidoreductase situated in the thylakoid membranes of cyanobacteria and chloroplasts. In land plants, the PSII core complex consists of the four large intrinsic subunits, D1 (PsbA), D2 (PsbD), CP43 (PsbC), and CP47 (PsbB); 14 low-molecular-mass membrane-spanning subunits, PsbE, PsbF, PsbH, PsbI, PsbJ, PsbK, PsbL, PsbM, PsbTc, PsbW, PsbX, PsbY, PsbZ, and Psb30; and five extrinsic subunits, PsbO, PsbP, PsbQ, PsbR, and PsbTn (Pagliano *et al.*, 2013; Wei *et al.*, 2016; van Bezouwen *et al.*, 2017). The latter stabilize and shield the Mn_4_CaO_5_ cluster and are attached to the lumenal surface of the core complex to form the oxygen evolving complex. PSII core monomers assemble into dimers to which, at both sides, light harvesting proteins (LHCII) bind to form PSII supercomplexes. In land plants, a PSII dimer binds two each of the monomeric minor LHCII proteins CP24 (LHCB6), CP26 (LHCB5), and CP29 (LHCB4) in addition to up to four major LHCII heterotrimers (Caffarri *et al.*, 2009; Kouřil *et al.*, 2011). In *Chlamydomonas reinhardtii*, lacking CP24, a PSII dimer binds two each of the CP26 and CP29 monomers as well as up to six major LHCII heterotrimers (Tokutsu *et al.*, 2012).

The individual steps leading to the assembly of PSII core complexes are well understood (Komenda *et al.*, 2012; Shi *et al.*, 2012; Nickelsen and Rengstl, 2013; Lu, 2016; Plochinger *et al.*, 2016). PSII assembly starts with the synthesis of the α- and β-subunits (PsbE and PsbF) of cytochrome *b*_559_, which can accumulate in the membrane in the absence of the D1 and D2 proteins and that interact with newly synthesized D2 (Morais *et al.*, 1998; Müller and Eichacker, 1999; Komenda *et al.*, 2004). A newly synthesized D1 precursor first interacts with PsbI formed ahead, followed by their assembly with the D2–cytochrome *b*_559_ complex into the reaction center (RC) (Dobáková *et al.*, 2007). Next follows the proteolytic processing of the D1 precursor at its C-terminus (Anbudurai *et al.*, 1994). Unlike in higher plants, the cyanobacterial D1 precursor is cleaved in two consecutive steps, with the primary cut occurring before and the final after CP47 assembly (Komenda *et al.*, 2007). Along with CP47, the low-molecular-mass subunits PsbH, PsbL, PsbM, PsbR, PsbTc, PsbX, and PsbY join to form the RC47 (or CP43-free PSII monomer) intermediate (Rokka *et al.*, 2005; Boehm *et al.*, 2012). The addition of CP43 with the small subunits PsbK, PsbZ, and Psb30 leads to the formation of PSII monomers (Sugimoto and Takahashi, 2003; Rokka *et al.*, 2005; Boehm *et al.*, 2011). During photoactivation, the Mn_4_CaO_5_ cluster is attached to the lumenal side of PSII monomers, followed by the PsbO, PsbP, and PSBQ proteins in chloroplasts (Bricker *et al.*, 2012). After dimerization of PSII monomers and attachment of LHCII trimers the assembly is complete and the supercomplex is transferred from stroma-exposed membranes to grana stacks (Tokutsu *et al.*, 2012; van Bezouwen *et al.*, 2017).

PSII assembly is facilitated by auxiliary factors that transiently bind to discrete assembly intermediates and are not part of the final complex. Many, but not all, of these auxiliary factors are conserved between cyanobacteria and chloroplasts (Nixon *et al.*, 2010; Nickelsen and Rengstl, 2013). Auxiliary factors involved in early steps of PSII *de novo* assembly include the membrane protein insertase Alb3 (Ossenbühl *et al.*, 2004; Göhre *et al.*, 2006), the TPR protein LPA1 (PratA in *Synechocystis*) that loads early PSII precomplexes with Mn^2+^ (Peng *et al.*, 2006; Stengel *et al.*, 2012), or HCF136 (YCF48 in *Syncechocystis*) that facilitates formation of the RC complex (Meurer *et al.*, 1998; Komenda *et al.*, 2008). Later steps of PSII assembly also require auxiliary factors like LOW PSII ACCUMULATION 2 (LPA2), which was proposed to assist in CP43 assembly (Chi *et al.*, 2012). Other auxiliary factors associated with PSII are involved in regulating the dynamics of PSII assembly states. Examples are MET1/TEF30, which interacts with PSII monomers and facilitates PSII supercomplex formation (Bhuiyan *et al.*, 2015; Muranaka *et al.*, 2016), and PSB33/LIL8, which controls the dynamics of LHCII (Fristedt *et al.*, 2015, 2017; Kato *et al.*, 2017). PSII can undergo repair after photodamage of the D1 protein. Here, many of the *de novo* assembly factors are involved also in the repair cycle, while others are specific for repair (Lu, 2016; Theis and Schroda, 2016).

The analysis of PSII assembly and the factors involved was greatly facilitated by the technique of blue-native (BN)–PAGE, which allows separating membrane protein complexes according to their size (Schägger and von Jagow, 1991; Schägger *et al.*, 1994). A follow-up technique is complexome profiling, where a lane of a BN gel is cut along the gradient into dozens of even slices and proteins therein are identified and quantified by mass spectrometry after tryptic in-gel digestion (Heide *et al.*, 2012; Heide and Wittig, 2013). By this, migration profiles are obtained for hundreds of proteins, and proteins with similar migration profiles are likely present in common protein complexes. This technique has been applied to several photosynthetic organisms, including Arabidopsis, *Physcomitrella patens*, *Chlamydomonas reinhardtii*, and five cyanobacterial species resulting in the Protein Co-Migration Database for photosynthetic organisms (PCoM) (Takabayashi *et al.*, 2017).

In this study we have identified the *Chlamydomonas* homolog of LPA2 and employed the complexome profiling technique to thylakoids isolated from wild type and a *lpa2* mutant to provide new insights into the function of LPA2 in PSII biogenesis. Moreover, the distinct migration profiles of PSII core subunits in wild type and *lpa2* mutant allowed the identification of novel proteins potentially associated with PSII based on their co-migration with PSII core subunits.

## Materials and methods

### Strains and cultivation conditions

*Chlamydomonas reinhardtii* wild type CC-4533 and *lpa2* mutant strain LMJ.RY0402.141537 from the *Chlamydomonas* library project (Li *et al.*, 2016) were obtained from the *Chlamydomonas* Resource Center. The *lpa2* mutant was used as recipient strain for transformation with plasmid pMBS683 to generate complemented lines c10 and c11, and pMBS684 to generate the complemented line cHA. Transformation was done by electroporation. Unless indicated otherwise, cultures were grown mixotrophically in TAP medium (Kropat *et al.*, 2011) on a rotatory shaker at 25 °C and ~30 µmol photons m^−2^ s^−1^. Cell densities were determined using a Z2 Coulter Counter (Beckman Coulter). For growth tests, cells were grown to a density of 3–5×10^6^ cells ml^−1^ and diluted in TAP medium or high salt medium (HSM) such that 20 µl contained 10^4^, 10^3^, or 10^2^ cells. Twenty microliters of each dilution were spotted onto agar plates containing TAP medium or HSM medium and incubated in low light (30 µmol photons m^−2^ s^−1^) for 72 h, high light (600 µmol photons m^−2^ s^−1^) for 72 h, or in the dark for 96 h. HSM was prepared according to Sueoka (1960), but using the trace solutions from Kropat *et al.* (2011).

### Cloning of the construct for complementing the *lpa2* mutant

The *LPA2* coding sequence was amplified by PCR from *Chlamydomonas* cDNA with primers 5′-aagaagAcAGAATGCAGACCTGCTTTTCA-3′ and 5′- ttgaagacttcgaaccCTGCTTCTGGATCTGTCCGGGC-3′ (where lowercase letters indicate altered bases to introduce *Bbs*I recognition sites). The resulting 552 bp PCR product was cloned into the recipient plasmid pAGM1287 (Weber *et al.*, 2011) by restriction with *Bbs*I and ligation with T4 ligase, resulting in the level 0 construct pMBS680. This level 0 part was then complemented with level 0 parts (pCM) from the *Chlamydomonas* MoClo toolkit (Crozet *et al.*, 2018) to fill the respective positions in level 1 modules as follows: A1–B2, pCM0-020 (*HSP70A-RBCS2* promoter + 5′UTR); B5, pCM0-100 (3×HA) or pCM0-101 (MultiStop); B6, pCM0-119 (*RPL23* 3′UTR). The *HSP70A-RBCS2* fusion promoter used here contains −467 bp of *HSP70A* sequences upstream from the start codon in optimal spacing with respect to the *RBCS2* promoter (Lodha *et al.*, 2008; Strenkert *et al.*, 2013). The level 0 parts and destination vector pICH47742 (Weber *et al.*, 2011) were combined with *Bsa*I and T4 DNA ligase and directionally assembled into level 1 modules pMBS682 (3×HA) and pMBS681 (MultiStop), which were then combined with pCM1-01 (level 1 module with the *aadA* gene conferring resistance to spectinomycin) from the *Chlamydomonas* MoClo kit, with plasmid pICH41744 containing the proper end-linker, and with destination vector pAGM4673 (Weber *et al.*, 2011), digested with *Bbs*I, and ligated to yield level 2 devices pMBS684 and pMBS683, respectively.

### RNA extraction and qRT-PCR

After centrifugation at 4300 *g* for 2 min, 10^8^ cells were harvested and then resuspended in lysis buffer (0.6 M NaCl, 0.1 M Tris–HCl, pH 8, 10 mM EDTA, 4% SDS), snap-frozen in liquid N_2_ and stored at −80 °C until further use. For RNA extraction, samples were incubated at 65 °C for 10 min, supplemented with 2 M KCl, incubated for 15 min on ice and centrifuged at 16 500 *g* for 15 min. Two extraction steps followed with phenol/chloroform/isoamylalcohol (25:24:1) and chloroform/isoamylalcohol (24:1) and RNA was precipitated over night at 4 °C in 8 M LiCl. The precipitate was collected by centrifugation at 16,500 *g* for 15 min, resuspended in RNase free H_2_O and supplemented with 3 M Na-acetate, pH 5.2. The RNA was precipitated for 45 min in 100% ethanol on ice and washed with 70% ethanol. After a further centrifugation step, the pelleted and dried RNA was resuspended in RNase free H_2_O, the concentration was determined spectrophotometrically using a NanoDrop 2000 (Thermo Scientific) and the RNA quality was confirmed using agarose gel electrophoresis. DNA contamination was removed with RNase-free Turbo DNase (Ambion) and complementary DNA (cDNA) synthesis was performed using the M-MLV reverse transcriptase (Promega), deoxynucleotide triphosphates, and oligo-d(T)_18_ primers. Quantitative reverse transcription-PCR (qRT-PCR) was performed using the StepOnePlus RT-PCR system (Thermo Fisher Scientific) and the 5× HOT FIREPol EvaGreen qPCR Supermix kit from Solis BioDyne. Each reaction contained the manufacturer’s master mix, 150 nM of each primer and cDNA corresponding to 10 ng of input RNA in the reverse transcription reaction. The reaction conditions were as follows: 95 °C for 10 min, followed by cycles of 95 °C for 15 s, 65 °C for 20 s, and 72 °C for 20 s, up to a total of 40 cycles. Primers used for the amplification of the *LPA2* transcript were 5′-GGGCTTTGGTTCAGAGACGG-3′ and 5′-TGCGTTCACCTTGACCTTGG-3′. Housekeeping controls used were *CBLP2* with primers 5′-GCCACACCGAGTGGGTGTCGT GCG-3′ and 5′-CCTTGCCGCCCGAGGCGCACAGCG-3′ and *TUB1* with primers 5′-CCCCCGCCTGCACTTCTTC-3′ and 5′-GTCGGCGG CGCACATCAT-3′.

### Protein analyses

Cells were harvested by centrifugation, resuspended in sample buffer containing 75 mM Tris–HCl, pH 6.8, 2% (w/v) SDS and 10% (v/v) glycerol, boiled at 95 °C and centrifuged. After quantification of protein concentrations according to Bradford (1976), Laemmli buffer (Laemmli, 1970) was added to samples and SDS-PAGE and semi-dry western blotting were performed as described previously (Liu *et al.*, 2005). Antisera used were against D1 (Agrisera AS05 084), CP43 (Agrisera AS11 1787), CP47 (Agrisera AS04 038), PsaA (Agrisera AS06 172), PSAN (M. Schroda), CytF (Pierre and Popot, 1993), CGE1 (Schroda *et al.*, 2001), LHCBM9 (M. Schroda), CF1β (Lemaire and Wollman, 1989), RPL1 (Ries *et al.*, 2017) and the HA-tag (Sigma-Aldrich H3663). Anti-rabbit IgG–horseradish peroxidase (HRP) (Sigma-Aldrich) and anti-mouse IgG–HRP (Santa Cruz Biotechnology sc-2031) were used as secondary antibodies.

BN-PAGE with whole cell proteins was carried out as described previously (Schägger and von Jagow, 1991) with minor modifications. Briefly, 10^8^ cells were pelleted, washed with 750 µl of TMK buffer (10 mM Tris–HCl, pH 6.8, 10 mM MgCl_2_, 20 mM KCl) and resuspended in 350 µl of ACA buffer (750 mM ε-aminocaproic acid, 50 mM Bis–Tris–HCl, pH 7.0, 0.5 mM EDTA) supplemented with 0.25× protease inhibitor (Roche). Cells were then broken by sonication and starch and cell debris were removed by centrifugation at 300 *g* for 5 min at 4 °C. Cell lysates (equivalent to 0.8 µg µl^−1^ of protein) were solubilized with 1% *n*-dodecyl α-D-maltoside (α -DDM) for 20 min on ice and in darkness. Insolubilized material was pelleted at 18 500 *g* for 10 min at 4 °C and the supernatant was supplemented with loading buffer (0.5 M ε-aminocaproic acid, 75% glycerol, 2.5% Serva Blue G-250 (Carl Roth)). After three cycles of centrifugation at 18 500 *g* for 10 min at 4 °C and transferring the supernatant to a fresh tube, samples were loaded on a 4–15% BN acrylamide gel.

The isolation of thylakoids was performed according to Chua and Bennoun (1975) with minor modifications. Briefly, ~2×10^9^ cells were pelleted and washed with 25 mM HEPES–KOH, pH 7.5, 5 mM MgCl_2_ and 0.3 M sucrose, before resuspending in the same buffer supplemented with protease inhibitor (Roche). Cells were then lysed using a BioNebulizer (Glas-Col) with an operating N_2_ pressure of 1.5–2 bar. After centrifugation at 3800 *g* for 10 min, the pellet was washed with 5 mM HEPES–KOH, pH 7.5, 1 mM EDTA and 0.3 M sucrose before resuspending in 5 mM HEPES–KOH, pH 7.5, 1 mM EDTA and 1.8 M sucrose. After a sucrose step gradient (0.5 M, 1.3 M, 1.8 M) centrifugation at 73 000 *g* for 1 h, intact thylakoids, floating between the 1.3 M and 1.8 M layers, were collected and diluted with 5 mM HEPES–KOH, pH 7.5 and 1 mM EDTA. After another centrifugation at 18 000 *g* for 1 h, the pellet was resuspended in small volumes of the same buffer.

For pulse labelling experiments, cells in the exponential growth phase (2×10^6^ cells ml^−1^) from a 100-ml culture were harvested by centrifugation, washed with minimum medium and re-suspended in 1/20th volume of minimum medium. Cells were allowed to recover and to deplete their intracellular carbon pool for 1.5 h under dim light (20 µmol photons m^−2^ s^−1^) and strong aeration at 25°C; 10 µM cycloheximide and 10 µCi ml^−1^ Na-[^14^C]acetate (PerkinElmer: 56.6 mCi mM^−1^) were then added to the culture. After 7 min the pulse was stopped by transferring the cells into 35 ml of ice-cold TAP medium containing 50 mM non-radioactive acetate. Cell samples, collected immediately after centrifugation at 4°C, were resuspended in ice-cold 0.1 M dithiothreitol and 0.1 M Na_2_CO_3_, frozen in liquid nitrogen, and kept at –80°C until analysis. For chase experiments, pulse-labelled cells were diluted in 35 ml of TAP medium containing 50 mM non-radioactive acetate and 250 µg ml^−1^ chloramphenicol at 25°C and further incubated in this medium for 20 and 60 min. Cells were then collected after centrifugation at 4°C and treated as above.

### In-gel digestion and mass spectrometry

Coomassie stained BN-PAGE gel pieces were destained by repeated cycles of washing with 40 mM NH_4_HCO_3_ for 5 min and incubating in 70% acetonitrile for 15 min, until they were colorless. They were then dehydrated completely by adding 100% acetonitrile for 5 min and dried under vacuum. Samples were then digested by covering the gel pieces in 10 ng μl^−1^ trypsin in 40 mM NH_4_HCO_3_ and incubating them overnight at 37 °C, before first hydrophilic peptides were extracted with 10% acetonitrile and 2% formic acid for 20 min and afterwards all other tryptic peptides were extracted with 60% acetonitrile and 1% formic acid. Samples were then desalted according to (Rappsilber *et al.* (2007)). Mass spectrometry was performed basically as described previously (Hammel *et al.*, 2018). For peptide separation, a HPLC flow rate of 4 μl min^−1^ and 21 min gradients from 2% to 33% HPLC buffer B were employed (buffer A: 2% acetonitrile, 0.1% formic acid; buffer B: 90% acetonitrile, 0.1% formic acid). MS1 spectra (350 *m*/*z* to 1250 *m*/*z*) were recorded for 250 ms and 25 MS/MS scans (100 *m*/*z* to 1500 *m*/*z*) were triggered in high sensitivity mode with a dwell time of 50 ms resulting in a total cycle time of 1550 ms. Analysed precursors were excluded for 5 s, and singly charged precursors or precursors with a response below 500 cps were excluded completely from MS/MS analysis.

### Evaluation of MS data

The analysis of MS runs was performed using MaxQuant version 1.6.0.1 (Cox and Mann, 2008). Library generation for peptide spectrum matching was based on *Chlamydomonas reinhardtii* genome release 5.5 (Merchant *et al.*, 2007) including chloroplast and mitochondrial proteins. Oxidation of methionine and acetylation of the N-terminus were considered as peptide modifications. Maximal missed cleavages were set to 3 and peptide length to 6 amino acids, the maximal mass to 6000 Da. Thresholds for peptide spectrum matching and protein identification were set by a false discovery rate of 0.01. The mass spectrometry proteomics data have been deposited to the ProteomeXchange Consortium via the PRIDE (Perez-Riverol *et al.*, 2019) partner repository with the dataset identifier PXD023443. Total protein group intensities varied between samples. For sample normalization, the total ion intensity sum of every protein and gel slice was calculated for each of the six samples (three wild type and three mutant). For every sample, a correction factor was determined by dividing every total ion intensity sum by the average total intensity sum. Subsequently, every intensity value was divided by its corresponding correction factor, to equalize all total ion intensity sums. For further analysis, proteins identified by non-proteotypic peptides were discarded. Protein identifiers were annotated with MapMan ontology terms, Gene Ontology (GO) terms, and proposed subcellular localization using the Functional Annotator (FATool available at http://iomiqsweb1.bio.uni-kl.de:8015/). Welch’s test was performed for each protein by considering the sums of all 36 normalized slice intensities for each sample and testing three wild type sums against three mutant sums. The distance of the average migration profiles for every protein was calculated as the Euclidean distance between wild type and mutant. To adjust for amplitude-introduced bias, each distance was divided by the maximal average intensity of wild type or mutant, respectively. Data normalization and analysis were performed using FSharp.Stats (https://github.com/CSBiology/FSharp.Stats). The migration profiles were visualized using Plotly.NET (https://github.com/muehlhaus/FSharp.Plotly) and NOVA software (Giese *et al.*, 2015).

### Cell fractionation

Crude fractionation was performed to separate soluble and membrane proteins. Cells (2×10^7^) were pelleted and resuspended in 1 ml of lysis buffer (10 mM Tris–HCl, pH 8.0, 1 mM EDTA) including 0.25× protease inhibitor (Roche)). A 200 µl whole cell aliquot was taken and supplemented with 50 µl of sample buffer (225 mM Tris–HCl, pH 6.8, 50% glycerol, 5% SDS, 0.25 M dithiothreitol, 0.05% bromophenol blue). The remaining solution was frozen in liquid nitrogen and thawed at room temperature for four cycles. After centrifugation at 21 000 *g* for 20 min, the supernatant, containing soluble proteins, was collected. The pellet fraction was resuspended in lysis buffer. Sample buffer was added to both protein extracts prior to boiling at 95 °C and protein separation via SDS-PAGE.

### Chlorophyll fluorescence measurements

Chlorophyll fluorescence was measured using a pulse amplitude-modulated Mini-PAM fluorometer (Mini-PAM, H. Walz, Effeltrich, Germany) essentially according to the manufacturer’s protocol after 3 min of dark adaptation (1 s saturating pulse of 6000 μmol photons m^−2^ s^−1^, gain = 4). Fluorescence emission spectra at 77 K were measured with an in-house built set-up. Samples were immersed in liquid nitrogen and excited with a LED source (LS-450, Ocean Optics—blue LED, 450 nm). The emission spectra were recorded using a charge-coupled device (CCD) spectrophotometer (QE6500, Ocean Optics). State 1 was achieved by placing the cells in oxidizing conditions upon illumination in the presence of the PSII inhibitor 3-(3,4-dichlorophenyl)-1,1-dimethylurea (DCMU; 10 μM). State 2 was achieved by placing the cells in reducing conditions (anoxia) upon addition of glucose (20 mM) and glucose oxidase (30 u ml^−1^) to the cultures in the dark for 20 min.

### Transmission electron microscopy

Cells were collected and washed in 100 mM sodium cacodylate at pH 7.2. Afterwards, cells were fixed in 100 mM sodium cacodylate containing 2.5% glutaraldehyde and 4% formaldehyde at pH 7.2 at room temperature. The buffer was exchanged after 20, 60, and 120 min. All other steps were done as described previously (Nordhues *et al.*, 2012). Samples were analysed with a JEM-2100 (JEOL) transmission electron microscope (operated at 80 kV). Micrographs were taken using a 4080×4080-pixel CCD camera (UltraScan 4000; Gatan) and Gatan DigitalMicrograph software (version 1.70.16).

### Sequence alignments and predictions

Putative chloroplast transit peptides of LPA2 homologs were predicted with ChloroP (Emanuelsson *et al.*, 1999) and putative transmembrane helices with HMMTOP (Tusnády and Simon, 2001). Sequence alignments were done with CLUSTALW (https://www.genome.jp/tools-bin/clustalw) and displayed with GeneDoc.

### Extraction of *Chlamydomonas* genomic DNA

Five milliliters of exponentially growing cells were pelleted and resuspended in 250 μl water; 250 μl 2× lysis buffer (20 mM Tris–HCl, 40 mM Na_2_EDTA, 6% (w/v) SDS) and 60 μg proteinase K (NEB) were added and incubated under agitation for 2 h at 55 °C. The lysate was supplemented with 80.9 μl of 5 M NaCl and mixed by vortexing. After the addition of 70 μl prewarmed cetyltrimethylammonium bromide (CTAB)/NaCl (2% (w/v) CTAB; 1.4 M NaCl) lysates were vortexed and incubated under agitation for 10 min at 65°C. Nucleic acids were extracted twice by the addition of one volume phenol/chloroform/isoamyl alcohol (25:24:1), and once by the addition of one volume chloroform/isoamyl alcohol (24:1) followed by vortexing and centrifugation for 5 min at 18 000 *g* and 4°C. Nucleic acids were precipitated by adding an equal volume isopropanol. Finally, the pellet was resuspended in TE buffer (10 mM Tris–HCl, pH 8; 1 mM EDTA; 1 μg μl^−1^ RNase).

### Phylogenetic analyses

Phylogenetic analyses were performed using the Phylogeny.fr pipeline (Dereeper *et al.*, 2008) implementing algorithms T-Coffee (Notredame *et al.*, 2000), BioNJ (Gascuel, 1997), and TreeDyn (Chevenet *et al.*, 2006).

## Results

### The *lpa2* mutant accumulates hardly any PSII supercomplexes, shows impaired growth, and is sensitive to high light intensities

**Fig. 1.**
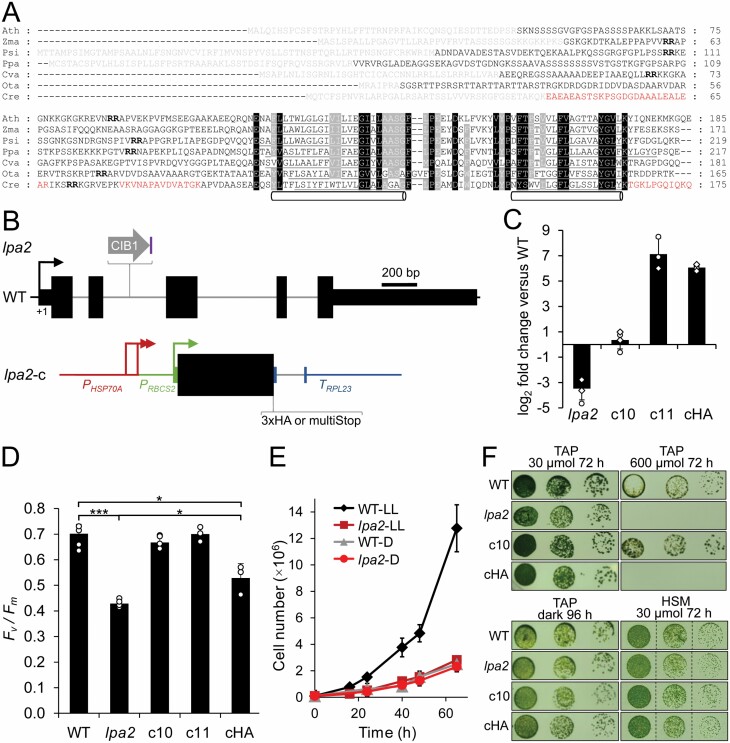
Phenotypes of the *lpa2* mutant compared with wild type and complemented lines. (A) Alignment of LPA2 amino acid sequences from different organisms. Predicted chloroplast transit peptides are shown in gray; predicted transmembrane helices are underlined and indicated with pipes. Peptides identified by mass spectrometry are in red letters, twin-arginines in bold letters. Ath, Arabidopsis (AT5G51545); Zma, *Zea mays* (NP_001145487); Psi, *Picea sitchensis* (ABK23742); Ppa, *Physcomitrella patens* (XP_024366975); Cva, *Chlorella variabilis* (XP_005849843); Ota, *Ostreococcus tauri* (XP_003084445); Cre, *Chlamydomonas reinhardtii* (Cre02.g105650). (B) Structure of the *Chlamydomonas LPA2* gene, insertion site of the CIB1 cassette in the *lpa2* mutant, and construct for complementation. Protein coding regions are shown as black boxes, untranslated regions as bars, and introns and promoter regions as thin lines. Arrows indicate transcriptional start sites. The purple box indicates a 165-bp fragment derived from the 19th intron of gene Cre13.g573450 in reverse orientation that has integrated with the CIB1 cassette. (C) qRT-PCR analysis of *LPA2* transcript accumulation in the *lpa2* mutant and two complemented lines expressing the *LPA2* cDNA without (c10, c11) or with a 3×HA coding region (cHA) relative to the wild type. Values are means from two independent biological replicates normalized against *CBLP2* (circles) or *TUB1* (diamonds). Error bars indicate SD. (D) Comparison of *F*_v_/*F*_m_ values. Values are averages from three to four independent experiments; error bars indicate SD. Significant differences were assessed via two-tailed, unpaired Student’s *t*-test with Bonferroni–Holm correction (*P<0.05, ***P<0.001). (E) Growth curves of wild type (WT) and *lpa2* mutant in low light (LL, 30 µmol photons m^−2^ s^−1^) and in the dark (D). Values are means from three independent experiments; error bars represent SD. (F) Analysis of the growth of 10^4^, 10^3^, and 10^2^ cells, shown from left to right, under the conditions indicated.

The PSII assembly factor LPA2 was claimed to be present only in plants and absent in *Chlamydomonas* and cyanobacteria, suggesting that it evolved after the divergence of the land plant lineage (Chi *et al.*, 2012). To test this, we performed database searches and could confirm the absence of genes encoding LPA2 homologs in cyanobacteria but could find a gene encoding a putative LPA2 homolog in *Chlamydomonas*. We analysed LPA2 homologs from land plants, moss, and green algae and found that they all share properties such as a chloroplast transit peptide followed by a sequence with a twin-arginine motif, and a highly conserved region with two predicted transmembrane domains (Fig. 1A).

**Fig. 2.**
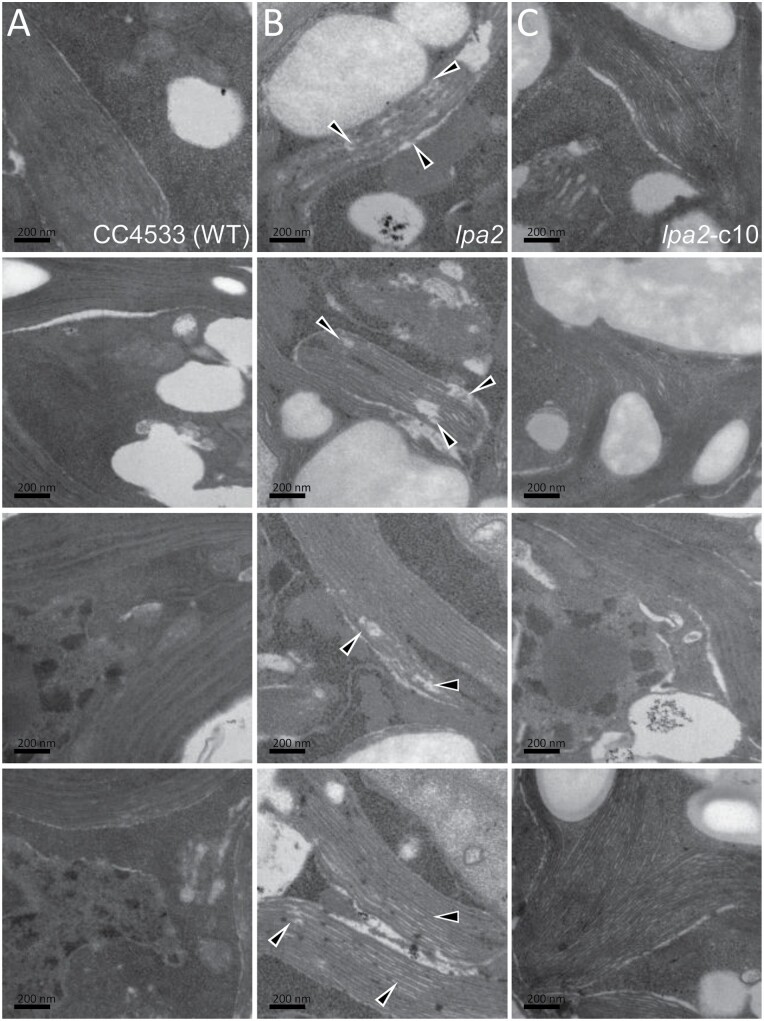
The *lpa2* mutant harbors aberrant structures within thylakoid membrane stacks. Electron microscopy images of wild type (A), *lpa2* mutant (B), and complemented line c10 (C). Triangles indicate aberrant regions within thylakoid membrane stacks in the *lpa2* mutant.

**Fig. 3.**
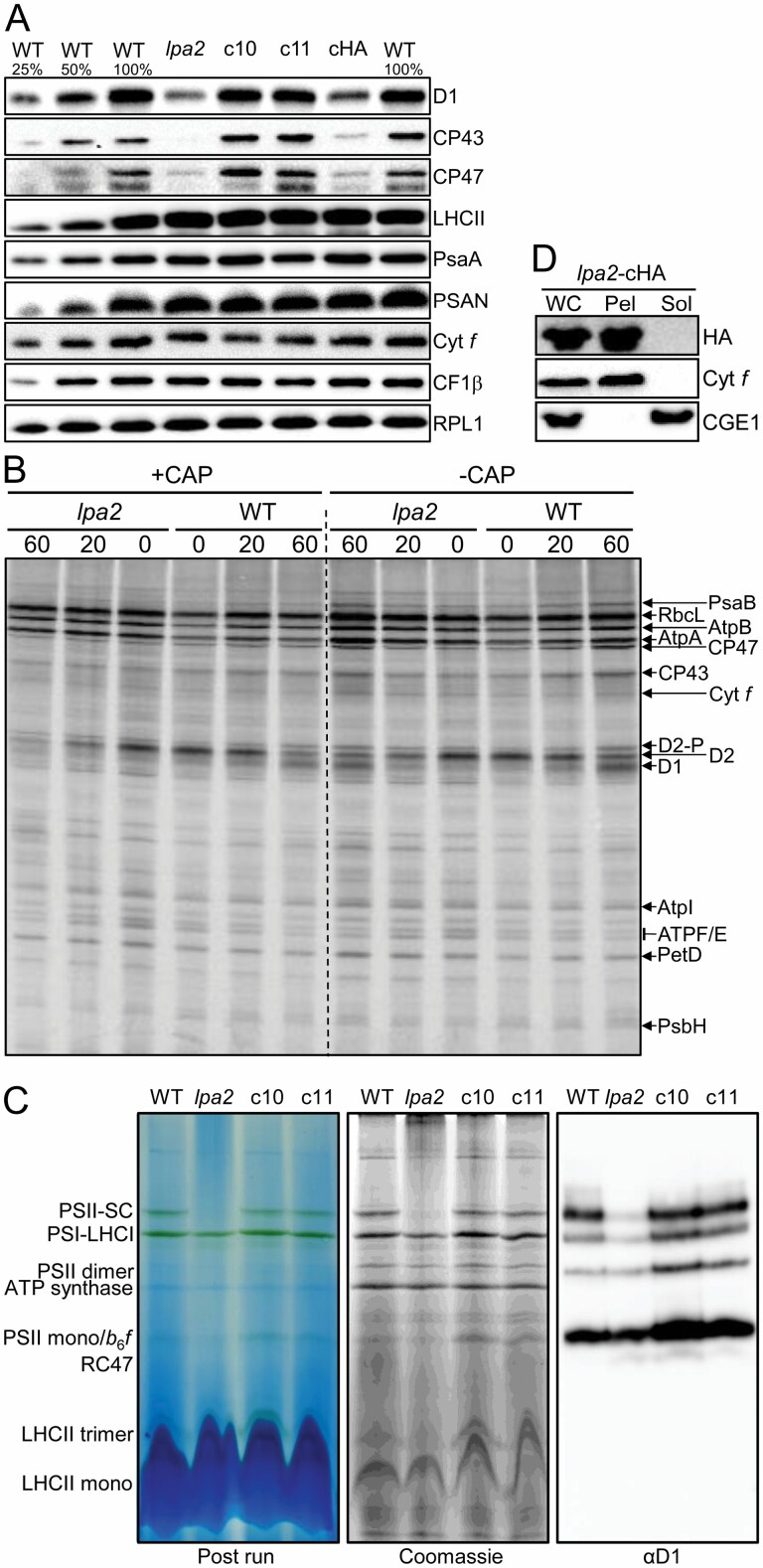
Analysis of protein accumulation, translation, complex assembly, and localization in *lpa2* mutant, wild type, and complemented lines. (A) Immunoblot analysis of the accumulation of subunits of the major thylakoid membrane protein complexes. PSII: D1, CP43, CP47, LHCII; PSI: PsaA, PSAN; Cyt *b*_6_*f* complex: Cyt *f*; ATP synthase: CF1β. Ribosomal protein RPL1 serves as loading control; 10 µg of whole-cell proteins (100%) were analysed. (B) Comparison of translation and stability of newly translated proteins between wild type (WT) and *lpa2* mutant. Cells were labelled with [^14^C]acetate for 7 min in the presence of cytosolic translation inhibitor cycloheximide (0) and chased with unlabeled acetate for 20 and 60 min in the presence or absence of chloramphenicol (CAP). Proteins were separated on a 12–18% SDS-urea gel and visualized by autoradiography. The assignment of the protein bands is based on mutant analyses (de Vitry *et al.*, 1989; Girard-Bascou *et al.*, 1992; Minai *et al.*, 2006). (C) BN-PAGE analysis. Thylakoid membranes were prepared from wild type (WT), *lpa2* mutant, and complemented lines c10 and c11, solubilized with α -DDM, and proteins separated on a 4–15% BN gel. Images show the gel after the run, after staining with Coomassie, and after immunoblotting and detection with an antibody against D1. SC, supercomplexes. (D) Crude fractionation of whole cells (WC) expressing LPA2–3×HA via freeze–thaw cycles and centrifugation into membrane enriched pellet (Pel) and soluble (Sol) fractions and analysis by immunoblotting. Integral membrane protein Cyt *f* and stromal CGE1 serve as markers for membrane and soluble fractions, respectively.

To test, whether the *Chlamydomonas* protein is a bona fide LPA2 homolog, we ordered a mutant from the *Chlamydomonas* library project (CLiP) (Li *et al.*, 2016) that contains the mutagenesis cassette in the second intron of the putative *LPA2* gene (Fig. 1B). The integration site of the cassette was verified by PCR on genomic DNA from the mutant (Supplementary Fig. S1). Sequencing of the amplified fragments revealed that the cassette had integrated together with a 165-bp fragment from a distant gene, probably derived from genomic DNA released from lysed cells (Zhang *et al.*, 2014). Although the cassette had inserted into an intron, expression of the putative *LPA2* gene was reduced by ~11-fold in the mutant compared with the wild type as judged by qRT-PCR analysis (Fig. 1C). The mutant exhibited a strongly reduced *F*_v_/*F*_m_ value compared with the wild type (0.43 versus 0.69, Fig. 1D) and slower mixotrophic growth in liquid culture at low light intensities, while heterotrophic growth in the dark was indistinguishable from the wild type (Fig. 1E). On solid medium, growth of the mutant under mixotrophic and photoautotrophic conditions in low light was only mildly impaired, while heterotrophic growth in the dark was unimpaired when compared with the wild type (Fig. 1F). In contrast, the mutant was unable to grow in high light under mixotrophic conditions. As judged by light microscopy, there were no obvious morphological differences between mutant and wild type cells (Supplementary Fig. S2). However, electron microscopy revealed aberrant structures in thylakoid membrane stacks in the *lpa2* mutant that were not observed in the wild type (Fig. 2A, B). Western blot analysis revealed that the accumulation of PSII core subunits D1, CP43, and CP47 was strongly reduced in the mutant compared with the wild type, while LHCII and core subunits of PSI, the cytochrome *b*_6_*f* complex, and the ATP synthase appeared to accumulate normally (Fig. 3A). [^14^C]Acetate pulse-chase labeling revealed no big differences in translation rates or stability of newly synthesized chloroplast-encoded subunits of the major thylakoid membrane complexes between mutant and wild type (Fig. 3B). However, BN-PAGE analysis showed an abolished accumulation of PSII complexes, particularly of PSII supercomplexes, in the mutant compared with the wild type (Fig. 3C).

To ensure that the observed phenotypes in the mutant were caused by the inactivation of the putative *LPA2* locus, we generated two constructs based on the *LPA2* cDNA and genetic parts from the Modular Cloning toolbox (Crozet *et al.*, 2018) for complementation. The cDNA was driven by the *HSP70A-RBCS2* fusion promoter, terminated by the *RPL23* terminator, and translationally fused with parts encoding either multiple stop codons or a C-terminal 3×HA epitope (Fig. 1B). The two resulting level 1 modules were combined with an *aadA* cassette, transformed into the mutant, and resulting spectinomycin-resistant transformants were screened for restored accumulation of the D1 protein (multi-stop construct) or accumulation of HA-tagged LPA2 protein (Supplementary Fig. S3). Of 20 transformants screened for each construct, we identified four with fully restored D1 accumulation (multi stop) and one with detectable expression of the HA-tagged protein migrating in the SDS gel with an apparent molecular mass of 22.4 kDa. qRT-PCR revealed that multi-stop transformant c10 accumulated *LPA2* transcript to wild type levels, while multi-stop transformant c11 accumulated it at ~140-fold higher levels than the wild type (Fig. 1C). The transformant harboring the construct encoding HA-tagged LPA2 accumulated *LPA2* transcripts at ~70-fold higher levels than the wild type.

While the *F*_v_/*F*_m_ value was fully restored in multi-stop transformants c10 and c11; this was not the case for the transformant expressing HA-tagged LPA2 (cHA) (Fig. 1D). Nevertheless, the value was slightly but significantly higher than in the mutant. The slower growth and increased high light sensitivity phenotypes of the mutant were fully complemented in the multi-stop transformant c10 (and also c11), while no complementation was observed for the cHA transformant (Fig. 1F). Also, the aberrant structures in thylakoid membrane stacks were no longer observed in the multi-stop transformant c10 (Fig. 2C). Moreover, D1, CP43, and CP47 accumulated to wild type levels in transformants c10 and c11, while in cHA these proteins accumulated only to slightly higher levels than in the mutant (Fig. 3A). Apparently, the 3×HA epitope at the C-terminus interfered with full functionality of the LPA2 protein, as has been observed for other PSII assembly factors like TerC and ALB3 (Ossenbühl *et al.*, 2006; Schneider *et al.*, 2014). Given the location of the two predicted transmembrane helices to the very C-terminus of LPA2, we wondered, whether the 3×HA tag disturbed proper membrane integration. To test this, we subjected cHA cells to freeze–thaw cycles and centrifugation for a crude separation of membrane and soluble proteins and detected transgenic LPA2 protein with an HA antibody. As shown in Fig. 3D, the protein was detected exclusively in the pellet fraction. This indicates that the C-terminal 3×HA tag did not disturb proper membrane integration of LPA2, but apparently negatively impacted other aspects important for its functionality. The formation of PSII supercomplexes was fully restored in transformants c10 and c11 (Fig. 3C).

In summary, inactivation of the putative *Chlamydomonas LPA2* gene resulted in reduced accumulation of PSII core subunits and PSII supercomplexes, a reduced *F*_v_/*F*_m_ value, slower growth in low light in liquid cultures and increased sensitivity to high light. All these phenotypes could be complemented by expressing the wild type protein, indicating that they are linked to the inactivated gene. As these phenotypes are in line with the proposed function of LPA2 in PSII assembly in Arabidopsis (Chi *et al.*, 2012; Schneider *et al.*, 2014), we can conclude that we have identified the *Chlamydomonas LPA2* gene.

### Comparing the complexome profiles of the *lpa2* mutant and wild type reveals insights into the role of LPA2 in PSII assembly and compensatory responses to cope with its absence

**Fig. 4.**
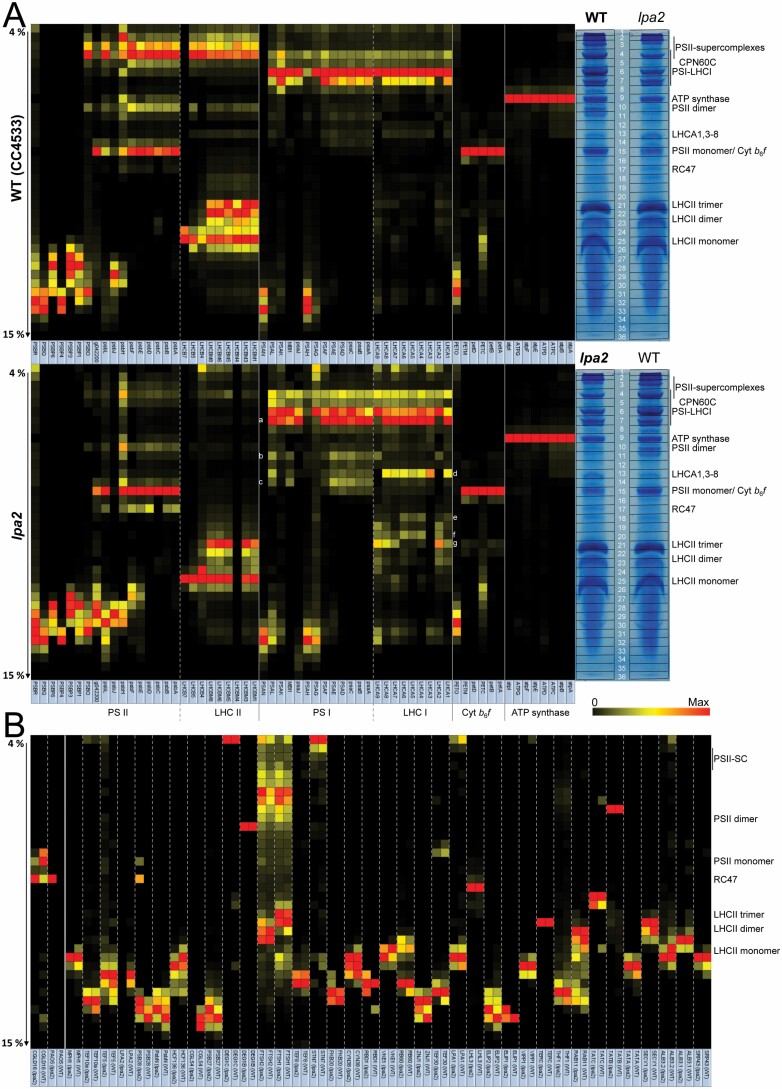
Complexome profiling of wild type and *lpa2* mutant. (A) Heat map showing the BN-PAGE migration profiles of subunits of the major thylakoid membrane protein complexes of wild type (WT, top panel) and *lpa2* mutant (bottom panel). Values for each protein are derived from averaged peptide ion intensities from three biological replicates and are normalized to the gel slice with highest intensities. The BN-PAGE lane of one replicate from WT and *lpa2* mutant is shown with the excised band corresponding to the heat map row. White letters (a–c) and (d–g) indicate different assembly states of PSI core and LHCI antenna proteins, respectively. The underlying data and the migration profiles for each protein are accessible in Supplementary Dataset S1. (B) Heat map showing the BN-PAGE migration profiles of known (right of solid line) and putatively new (left of solid line) auxiliary factors involved in PSII biogenesis, repair, and the regulation of PSII complex dynamics in wild type and *lpa2* mutant. The accession numbers of all proteins are listed in Supplementary Table S1.

To get more insights into the function of LPA2 in PSII biogenesis in *Chlamydomonas*, we made an in-depth comparison of the BN-PAGE migration profiles of thylakoid membrane proteins from wild type and *lpa2* mutant via complexome profiling (Heide *et al.*, 2012). To this end we isolated thylakoid membranes from wild type and *lpa2* mutant cells grown in low light (~30 µmol photons m^−2^ s^−1^) in three biological replicates. Thylakoid membranes were solubilized with α -DDM and protein complexes separated on a 4–15% BN gel (Supplementary Fig. S4). Each gel lane was cut into 36 slices and the resulting 216 slices were subjected to tryptic in-gel digestion and LC-MS/MS analysis. In total, 1734 proteins were identified. Extracted ion chromatograms were used for protein quantification followed by normalization based on total ion intensities per lane (although equal amounts of protein were loaded, there are differences between the replicates that make normalization necessary (Supplementary Fig. S4)). Ion intensity profiles for each protein along the BN gel run are displayed in the Excel table of Supplementary Dataset S1. The migration profiles of all identified proteins of wild type and *lpa2* mutant, clustered according to their migration behavior, are shown in Supplementary Dataset S2 as heat maps. The profiles for proteins belonging to the major thylakoid membrane complexes from wild type and *lpa2* mutant are shown as heat maps in Fig. 4. Missing subunits, such as PsbI, did not give rise to detectable peptides because peptides are too small, too large, too hydrophobic, or contain post-translational modifications other than methionine oxidation or *N*-acetylation.

Eight subunits of the ATP synthase were identified, and albeit their median abundance was ~29% higher in the *lpa2* mutant than in the wild type (Table 1), there was no difference in their migration patterns (Fig. 4A). Six subunits of the cytochrome *b*_6_*f* complex were identified. They were about equally abundant in wild type and *lpa2* mutant and displayed the same migration behavior (Table 1; Fig. 4A). The PETO protein was not associated with the cytochrome *b*_6_*f* complex, corroborating earlier findings by Takahashi *et al.* (2016). Interestingly, a substantial fraction of the Rieske iron–sulfur protein migrated as unassembled protein both in wild type and *lpa2* mutant, while all other subunits were quantitatively assembled into the complex.

**Table 1.** Ratios of subunit abundances between *lpa2* mutant and wild type

ATP synthase		Cytochrome *b*_6_*f*		Photosystem II		Photosystem I	
Subunit	Ratio lpa2/WT	Subunit	Ratio lpa2/WT	Subunit	Ratio lpa2/WT	Subunit	Ratio lpa2/WT
AtpA	1.27	PetA	1.01	PsbA (D1)	0.29	PsaA	0.93
AtpB	1.30	PetB	0.93	PsbB (CP47)	0.29	PsaB	0.71
ATPC	1.28	PETC	1.09	PsbC (CP43)	0.23	PsaC	0.88
ATPD	1.08	PetD	0.68	PsbD (D2)	0.30	PSAD	1.06
AtpE	1.52	PETM	1.52	PsbE	0.49	PSAE	1.15
AtpF	1.52	PETO	0.96	PsbF	0.55	PSAF	1.03
ATPG	1.28	**Median**	**0.99**	PsbH	0.67	PSAG	0.70
AtpI	1.72			PsbJ	2.17	PSAH	1.04
**Median**	**1.29**			PsbL	0.84	PsaJ	0.92
				PBA1	0.94	PSAK	1.35
				**Median**	**0.52**	PSAL	0.82
				PSBO	0.55	PSAN	1.25
				PSBP1	0.61	**Median**	**0.98**
				PSBP2	0.46	LHCA1	1.10
				PSBP3	1.08	LHCA2	1.19
				PSBP4	0.81	LHCA3	0.88
				PSBP6	0.61	LHCA4	0.96
				PSBQ	0.54	LHCA5	1.04
				PSBR	0.50	LHCA6	1.16
				**Median**	**0.58**	LHCA7	1.03
				LHCB4	1.20	LHCA8	1.10
				LHCB5	1.45	LHCA9	1.25
				LHCB7	1.79	**Median**	**1.10**
				LHCBM1	1.41		
				LHCBM3	1.25		
				LHCBM5	1.27		
				LHCBM6	1.27		
				LHCBM8	1.20		
				LHCBM9	0.94		
				**Median**	**1.27**		

Values are based on the summed ion intensities in all gel bands of three biological replicates each of wild type and mutant.

**Fig. 5.**
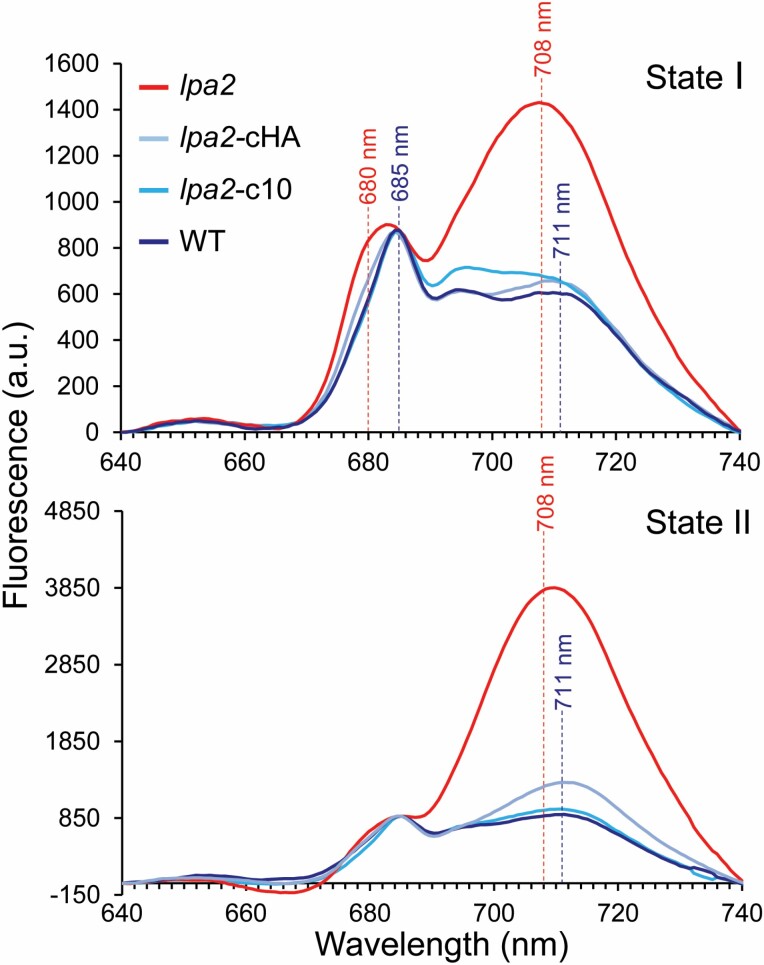
77K fluorescence spectra of wild type, *lpa2* mutant, and complemented lines either placed in state I by incubating cells under dim light in the presence of 10 µM DCMU or placed in state II by inducing anaerobiosis in the presence of 20 mM glucose and 30 units ml^−1^ glucose oxidase. Fluorescence emission spectra were normalized to the PSII emission peak at 685 nm for each strain.

In contrast to ATP synthase and cytochrome *b*_6_*f* complex, the composition of PSI complexes showed marked differences between wild type and mutant. The median abundance of PSI core subunits was unchanged and that of LHCI antenna proteins only slightly higher in the mutant than in the wild type (Table 1; Fig. 4A). However, the LHCI antenna was much more disconnected in the *lpa2* mutant, with LHCA1 and LHCA3–8 still forming a common complex that was even visible in the BN-PAGE gel, in addition to smaller assemblies (d–f in Fig. 4A). LHCA2 and LHCA9 were not present in this complex and accumulated only as smaller assemblies (g in Fig. 4A as the major one). As a result of the disconnected antenna, more PSI core complexes were observed at smaller apparent molecular mass in the mutant compared with the wild type (a–c in Fig. 4A). Moreover, more of the small PSI subunits PSAG, PSAL, PSAK, and PSAJ in this order were disconnected from the core complex and accumulated as unassembled subunits in the mutant. Most PSAH and all PSAN accumulated as unassembled subunits in both *lpa2* mutant and wild type, presumably because they lost connection to the PSI core during sample preparation or electrophoresis. To verify LHCI antenna disconnection from PSI in the *lpa2* mutant, we recorded 77K fluorescence spectra in wild type, *lpa2* mutant, and complemented lines placed in state I (to minimize energy transfer from LHCII to PSI) or in state II (to maximize energy transfer from LHCII to PSI) (Fig. 5). The blue shift of the PSI fluorescence peak (711 nm to 708 nm) in the *lpa2* mutant in state I is indicative of a disconnected PSI antenna (Tapie *et al.*, 1984; Hemme *et al.*, 2014).

**Fig. 6.**
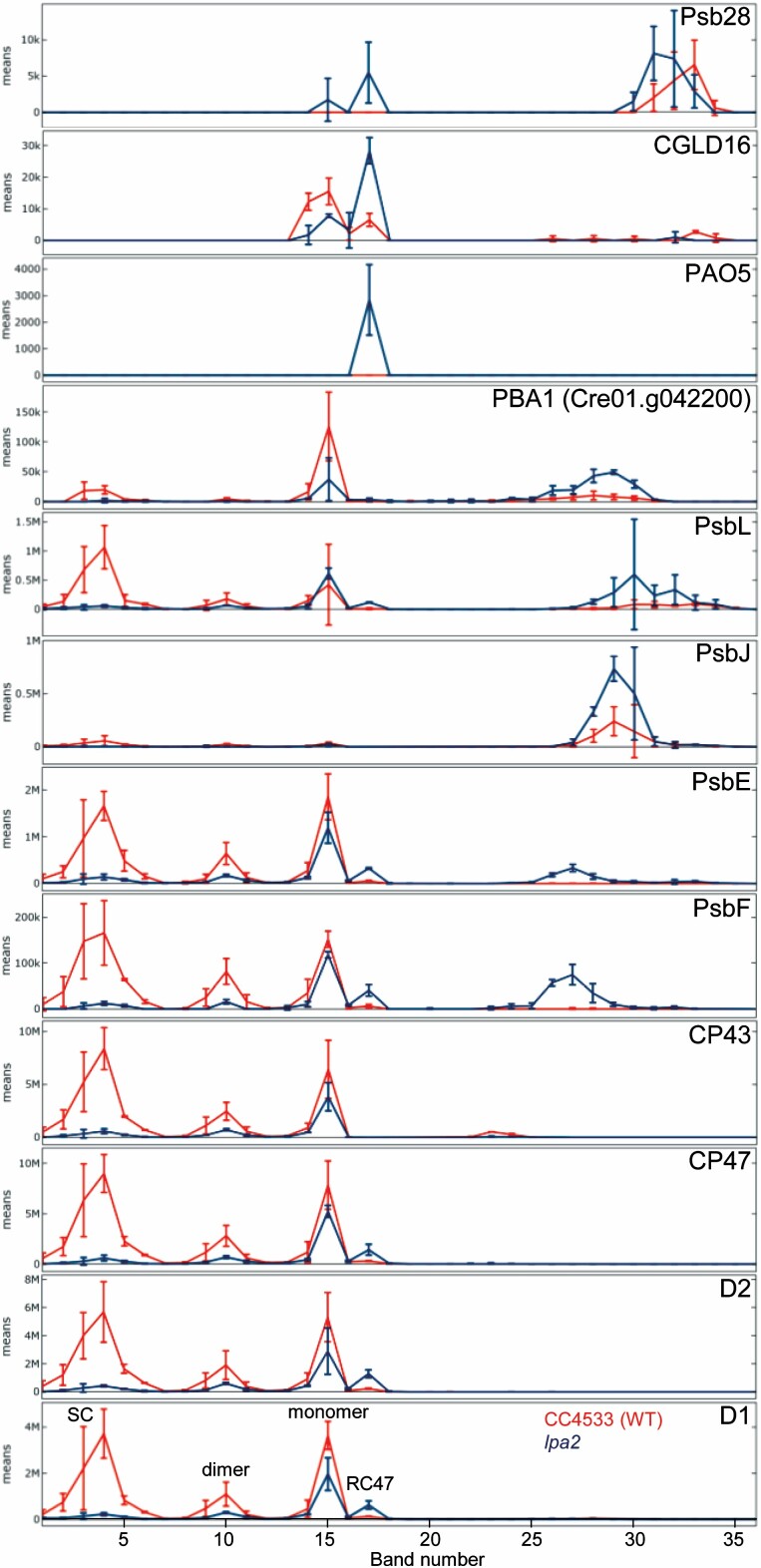
Comparison of BN-PAGE migration profiles of PSII core subunits and of putative novel proteins associated with PSII between wild type (red) and *lpa2* mutant (blue). Values for each protein are derived from averaged peptide ion intensities from three biological replicates. Error bars represent SD. Individual profiles from each replicate before and after normalization and statistical analyses can be accessed in Supplementary Dataset S1. SC, supercomplexes.

As expected, the most dramatic change between *lpa2* mutant and wild type was at the level of PSII supercomplexes, which accumulated only to ~7% of wild type levels in the mutant, as judged from the median abundance of the core subunits in the supercomplexes (Table 2; Figs 4A, 6). The abundance of dimers and monomers was reduced in the mutant as well, but not as markedly as that of the supercomplexes (~27% and ~60% of wild type levels, respectively). In contrast, about six times more RC47 accumulated in the mutant compared with the wild type, and large amounts of unassembled Cyt *b*_559_ (PsbE/F) accumulated only in the mutant. Also, much larger amounts of unassembled PsbJ and PsbL accumulated in the mutant compared with the wild type. We observed no RC, PsbI-D1, or PsbE/F-D2 assembly intermediates. Regarding the overall differences in abundance of PSII core subunits between mutant and wild type, the biggest difference was found for CP43, which accumulated only to ~23% of wild type levels, followed by CP47, D1, and D2 accumulating to ~30% of wild type levels in the *lpa2* mutant (Table 1). The small core subunits accumulated to between 49% (PsbE) and 84% (PsbL) of wild type levels. In contrast, PsbJ was more than 2-fold more abundant in the *lpa2* mutant than in the wild type.

**Table 2.** Ratios of subunit abundances in various PSII assembly states between *lpa2* mutant and wild type

	SC	Dimer	Monomer	RC47
PsbA (D1)	0.06	0.27	0.54	4.84
PsbB (CP47)	0.07	0.25	0.67	4.81
PsbC (CP43)	0.07	0.29	0.60	nd
PsbD (D2)	0.07	0.32	0.54	5.60
PsbE	0.09	0.27	0.64	6.05
PsbF	0.07	0.19	0.78	6.55
PsbJ	0.11	0.15	0.48	nd
PsbL	0.05	0.37	1.46	9.67
PBA1	0.10	0.14	0.30	6.28
**Median**	**0.07**	**0.27**	**0.60**	**6.05**

Values are based on the summed ion intensities in bands 4 (supercomplexes, SC), 10 (dimers), 15 (monomers), and 17 (RC47) from three biological replicates each of wild type and mutant. nd, not detected.

Except for PSBO, all other subunits involved in stabilizing/shielding the Mn_4_CaO_5_ cluster were found to migrate as unassembled subunits in both *lpa2* mutant and wild type, presumably because they got detached from PSII during sample preparation or electrophoresis. The median abundance of all subunits of the water-splitting complex reached ~58% of wild type levels. The median abundance of LHCII proteins was ~27% higher in the mutant than in the wild type and therefore, since they could not be assembled into PSII supercomplexes, there was a large pool of unassembled LHCII trimers and monomers in the mutant. The shoulder at 680 nm in the 77K fluorescence spectra in *lpa2* mutant cells placed in state I is indicative of a disconnected PSII antenna (Fig. 5). We could detect the recently identified LHCB7 protein with four transmembrane helices (Klimmek *et al.*, 2006). However, in contrast to LHCB4 (CP29) and LHCB5 (CP26), LHCB7 accumulated only in the unassembled form (Fig. 4A).

### The migration patterns of only a few known PSII auxiliary factors differ between *lpa2* mutant and wild type

We reasoned that changes in the migration patterns of known PSII auxiliary factors between *lpa2* mutant and wild type might reveal deeper insights into LPA2 function. Therefore, we started from a list of auxiliary factors compiled by Lu (2016) for Arabidopsis and searched for *Chlamydomonas* homologs that were detected in at least two replicates of mutant or wild type in the complexome profiling dataset (Supplementary Table S1). The migration profiles of the resulting 37 factors are displayed in the heat map shown in Fig. 4B. Most of the factors were found to migrate in the low-molecular-mass region below LHCII trimers. Here, LPA2 and LPA19 (CGL54), a factor involved in the C-terminal processing of D1 during assembly (Wei *et al.*, 2010), were completely missing in thylakoids from the mutant. Of the factors found in high-molecular-mass assemblies, we found five to display differences between mutant and wild type based on data from at least two replicates: ALB3.2, LPA1, TEF5, TEF30, and PSB28. ALB3.2, which is required for the insertion of photosystem core proteins into the thylakoid membrane (Göhre *et al.*, 2006), was found as unassembled protein and in a very large assembly hardly entering the gel. In the mutant, the balance between both forms was shifted to the unassembled protein. LPA1, an integral membrane protein required for proper PSII assembly (Peng *et al.*, 2006), also migrated as unassembled protein and in a very large assembly. The latter was less abundant in the mutant. TEF5 (PSB33 or LIL8 in Arabidopsis), involved in regulating LHCII dynamics (Fristedt *et al.*, 2015, 2017; Kato *et al.*, 2017), was more abundant in the mutant and migrated in several higher-molecular-mass assemblies only in the mutant. TEF30 (MET1 in Arabidopsis) interacts with PSII monomers and facilitates PSII supercomplex formation (Bhuiyan *et al.*, 2015; Muranaka *et al.*, 2016). We found less TEF30 to migrate with PSII monomers in the *lpa2* mutant, in line with the lower amount of PSII monomers in the mutant. Psb28 has been found to associate with PSII monomers, RC47, and non-assembled CP47 in *Synechocystis* and was suggested to play roles during PSII repair and during PSII and PSI biogenesis, by mediating the incorporation of chlorophyll (Dobáková *et al.*, 2009; Boehm *et al.*, 2012; Sakata *et al.*, 2013; Bečková *et al.*, 2017). We found PSB28 to co-migrate with PSII monomers and RC47 only in the *lpa2* mutant.

### Identification of novel proteins potentially associated with PSI and PSII

We reasoned that the characteristic, distinct migration profiles of PSII and PSI subunits in wild type and *lpa2* mutant might enable the identification of novel proteins interacting with the photosystems. We therefore searched among the 1734 proteins identified in our complexome profiling dataset for proteins that meet the following four criteria: (i) exhibit a migration profile resembling that of canonical PSI or PSII subunits in wild type and *lpa2* mutant; (ii) have been identified in at least two replicates in wild type or mutant; (iii) contain a putative chloroplast transit peptide; and (iv) are conserved in photosynthetic organisms. MBI1 met all four criteria to qualify as a protein potentially interacting with PSI. It co-migrated with PSI core subunits in wild type and *lpa2* mutant (Fig. 4A). MBI1 is an OPR protein that functions as a maturation factor of the *psbI* transcript and appears to be conserved only in the Chlamydomonadales (Wang *et al.*, 2015). Although MBI1 is a large protein (~138 kDa), we identified only a single peptide (AEAEAQRRLGLGLR) with a comparably low identification score but good ion intensity (Supplementary Dataset S3). We therefore cannot rule out that the underlying fragmentation spectrum derives from a modified peptide of a PSI core subunit that is isobaric with the assigned MBI1 peptide and produces similar fragment ions. Hence, this finding should be taken with great caution.

**Fig. 7.**
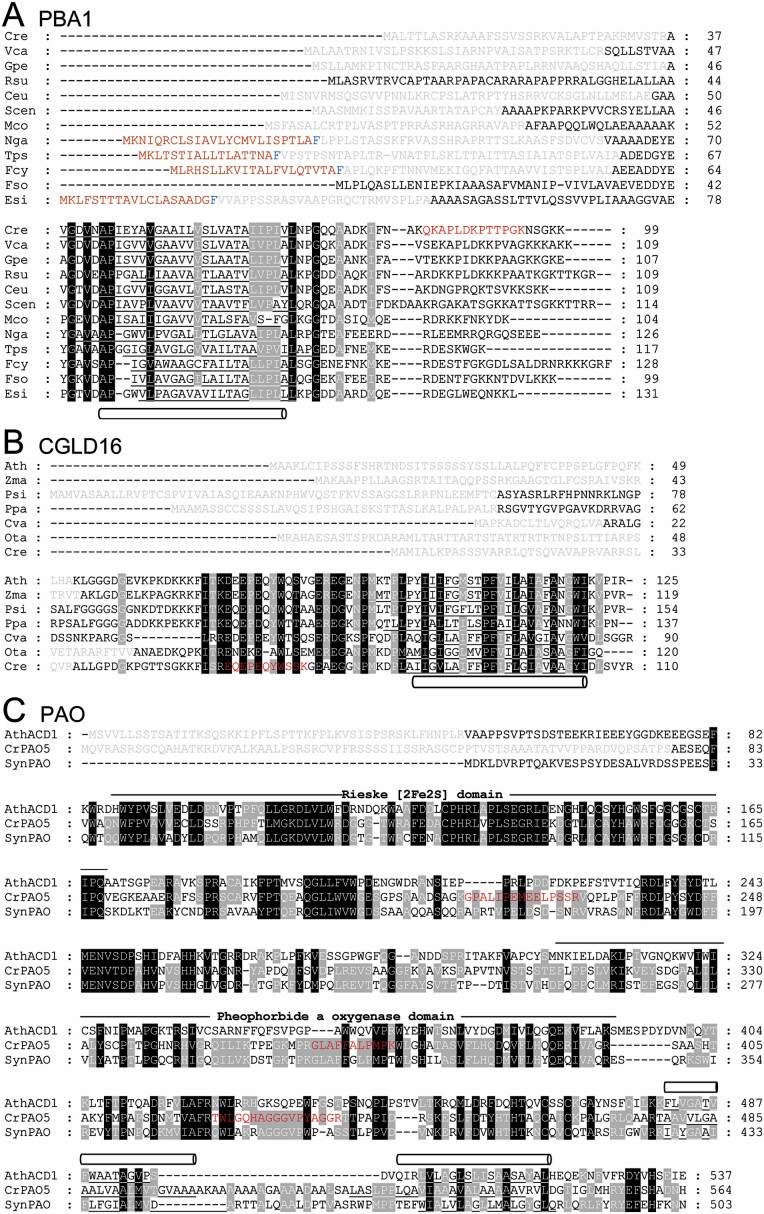
Alignment of amino acid sequences of putative novel PSII associated proteins from different organisms. Predicted chloroplast transit peptides are shown in grey and bipartite transit peptides from heterokonts are shown in brown with the conserved phenylalanine in blue (Kilian and Kroth, 2005). Predicted transmembrane helices are underlined and indicated with pipes. Peptides identified by mass spectrometry during complexome profiling are given in red letters. (A) PBA1 homologs. Chlorophytes (green algae): Cre, *Chlamydomonas reinhardtii* (Cre01.g042200); Vca, *Volvox carteri* (Vocar.0001s0632); Gpe, *Gonium pectorale* (KXZ55153); Rsu, *Raphidocelis subcapitata* (GBF92771); Ceu, *Chlamydomonas eustigma* (GAX81760); Scen, *Scenedesmus* sp. (KAF6255734); Mco, *Micractinium conductrix* (PSC75504). Eustigmatophytes: Nga, *Nannochloropsis gaditana* (EWM21362). Bacillariophytes (diatoms): Tps, *Thalassiosira pseudonana* (XP_002292489); Fcy, *Fragilariopsis cylindrus* (OEU08446); Fso, *Fistulifera solaris* (GAX25330). Phaeophytes (brown algae): Esi, *Ectocarpus siliculosus* (CBN78173). (B) CGLD16 homologs. Ath, Arabidopsis (AT2G05310); Zma, *Zea mays* (ACG27371); Psi, *Picea sitchensis* (ABR16811); Ppa, *Physcomitrella patens* (XP_024358733); Cva, *Chlorella variabilis* (XP_005848382); Ota, *Ostreococcus tauri* (XP_003078680); Cre, *Chlamydomonas reinhardtii* (Cre03.g151200). (C) ACD1 from Arabidopsis (AtACD1, AT3G44880) compared with PAO5 homologs from *Chlamydomonas reinhardtii* (Cre06.g278245) and *Synechococcus* sp. PCC 7335 (SynPAO, EDX87484).

The gene product of Cre01.g042200 met all four criteria to qualify as a protein potentially interacting with PSII (Figs 4A, 6). It co-migrated with PSII supercomplexes, dimers, RC47, and most pronounced, with monomers. Its abundance in these complexes was reduced in the *lpa2* mutant compared with the wild type, while its unassembled form was more abundant in the mutant. The protein encoded by Cre01.g042200 contains 99 amino acids of which the N-terminal 36 were predicted to represent a chloroplast transit peptide (Fig. 7A). The mature 63-amino-acid protein has a molecular mass of 6.4 kDa and contains a predicted transmembrane helix. Two peptides were identified with high identification scores and good ion intensities (Supplementary Dataset S3). We found homologs only in members of the green algae, brown algae, diatoms, and Eustigmatophytes (Fig. 7A). We named this protein PBA1 (putatively Photosystem B Associated 1).

The accumulation of the PSII assembly intermediate RC47 to ~6-fold higher levels in the *lpa2* mutant compared with the wild type (Table 2) opened the possibility of also identifying novel auxiliary factors in PSII assembly that specifically interact with RC47. To this end, we searched in the complexome profiling dataset for proteins that accumulated in band 17 of the *lpa2* mutant at much higher levels than in the wild type and meet criteria (ii)–(iv) introduced above. Three proteins meet all criteria. The first is PSB28, which accumulated in band 15 (PSII monomers) and 17 (RC47) only in the *lpa2* mutant, and as free protein in mutant and wild type (Figs 4B, 6). The second protein, encoded by Cre03.g151200 (CGLD16), co-migrated with PSII monomers and RC47. In the *lpa2* mutant, less of this protein was present in the monomer band and more in the RC47 band compared with the wild type. CGLD16 is conserved in the green lineage and diatoms. The N-terminal 36 of the overall 110 amino acids qualify as a chloroplast transit peptide, leaving a mature protein of 7.9 kDa that contains a predicted transmembrane domain at its C-terminus (Fig. 7B). Only one peptide was identified for CGLD16 with high identification score and good ion intensity (Supplementary Dataset S3). The last protein, encoded by Cre06.g278245 (PAO5), co-migrated only with RC47 and only in the *lpa2* mutant. It contains a predicted chloroplast transit peptide, a Rieske [2Fe–2S] domain, and a pheophorbide *a* oxygenase (PAO) domain (Fig. 7C). The mature protein has a molecular mass of 52 kDa. Three independent peptides were identified for PAO5 with rather low identification scores presumably due to the low precursor ion intensities (Supplementary Dataset S3). PAO5 is a member of a gene family with eight members in *Chlamydomonas*, while there are only three family members in Arabidopsis (Supplementary Fig. S5). Interestingly, the closest homolog of PAO5 is *Chlamydomonas* PAO6 followed by cyanobacterial PAOs, while there is no close homolog of PAO5 in Arabidopsis.

In summary, complexome profiling allowed deep quantitative insights into defects in PSII assembly and possible adaptive responses of the *lpa2* mutant and allowed the identification of potentially novel proteins interacting with the photosystems.

## Discussion

### LPA2 appears to catalyse a step that is essential for the assembly of PSII monomers into higher assembly states

We report here on the identification of an LPA2 homolog in *Chlamydomonas* and its functional analysis. We found LPA2 to be conserved in the green lineage but to be absent in cyanobacteria (Fig. 1A). All LPA2 homologs share two predicted transmembrane helices at their C-terminus, in accordance with the finding that the *Chlamydomonas* protein behaves like an integral membrane protein (Fig. 3D). All LPA2 homologs contain a twin-arginine motif in the sequence following the predicted cleavage site of the chloroplast transit peptide (Fig. 1A) but lack the highly hydrophobic residues at positions RR+2/3 and the A-X-A motif to qualify as precursors for TAT pathway-mediated import into the thylakoid lumen (Cline, 2015). Accordingly, there is no processing of this sequence, as judged from the identification of two tryptic peptides in this sequence by mass spectrometry (Fig. 1A; Supplementary Dataset S3). Possibly, the twin-arginine motif aids in directing the protein to the thylakoid membrane for insertion.

The *Chlamydomonas lpa2* mutant contains the mutagenesis cassette in the second intron of the *LPA2* gene (Fig. 1B), resulting in ~11-fold reduced *LPA2* transcript levels (Fig. 1C) and the virtual absence of LPA2 protein as judged by mass spectrometry: while LPA2 was detected with three peptides and good ion intensities in all replicates of the wild type, it was not detected in any of the replicates of the mutant (Supplementary Dataset S3). The *lpa2* mutant accumulates PSII core subunits D1, D2, and CP47 to ~30% of wild type levels and core subunit CP43 to only ~23% of wild type levels (Fig. 3A; Table 1). Small PSII subunits and subunits of the oxygen-evolving complex are less affected and accumulate to at least ~50% of wild type levels. Apparently, in line with the proposed role of LPA2 in assisting CP43 assembly (Chi *et al.*, 2012), CP43 is the most affected PSII subunit in the *lpa2* mutant and the RC47 intermediate accumulates to ~6-fold higher levels in the mutant than in the wild type (Figs 3A, 4A, 6; Tables 1, 2). However, neither the synthesis of CP43 or of any other PSII core subunit, nor their stability up to 60 min post-synthesis was markedly impaired (Fig. 3B). Moreover, the lack of CP43 or the synthesis of an unstable CP43 variant was shown to result in a fast and almost complete turnover of the other PSII core subunits, D1, D2, and CP47, in *Chlamydomonas* (Rochaix *et al.*, 1989). Therefore, it appears more likely that CP43 is initially assembled into monomers, accounting for normal synthesis and stability of the other core subunits, but in a way that renders further assembly states unstable. This scenario would explain why PSII monomers still accumulate to ~60% of wild type levels in the *lpa2* mutant, while dimers and supercomplexes reach only ~27% and ~7% of wild type levels, respectively. Accordingly, newly made PSII monomers might bounce back after the attempted assembly into dimers and supercomplexes and become subject to degradation. In this case, the largest part of the overaccumulating RC47 in the *lpa2* mutant results from the degradation of misassembled PSII monomers. Cyt *b*_559_ (PsbE/F), also accumulating to high levels in the *lpa2* mutant, might be a degradation product, too. The co-migration of pheophorbide *a* oxygenase PAO5 with RC47 in the mutant but not in the wild type might be indicative of active degradation of chlorophyll in RC47 in the mutant’s thylakoid membrane. A rather inefficient degradation of misassembled PSII monomers in the *lpa2* mutant appears supported by the finding that we found no changes in abundances or migration profiles of potentially involved major proteases in or at the thylakoid membranes, including FTSH1/2, DEG1C, and DEG1B (Fig. 4B) (Malnoë *et al.*, 2014; Theis *et al.*, 2019).

Although a role of LPA2 in the correct assembly of CP43 into PSII monomers well explains the observed phenotypes in the *lpa2* mutant, other interpretations are possible, since the inefficient assembly of PSII monomers into dimers and supercomplexes can have many causes. One possibility is that LPA2 is involved in the assembly of a small PSII core subunit, for example PsbI, the acceptor of newly made D1. In the *Chlamydomonas mbi1* mutant lacking PsbI, PSII assembly proceeds up to the monomer stage, but not beyond (Wang *et al.*, 2015). And in *Synechocystis* the absence of PsbI results in a destabilization of CP43 binding within PSII monomers and dimers (Dobáková *et al.*, 2007). Another possibility is that the *lpa2* mutant is impaired in the C-terminal processing of D1 because the absence of LPA2 in *Chlamydomonas* comes along with the absence of LPA19 (Fig. 4B), which facilitates this step in Arabidopsis (Wei *et al.*, 2010). LPA19 was detected with three peptides with average identification scores and low ion intensities in all three wild type replicates (Supplementary Dataset S3), and therefore it appears unlikely that we simply missed the protein in the mutant. The Arabidopsis *lpa19* mutant resembles the phenotype of the *Chlamydomonas lpa2* mutant regarding the lower accumulation of PSII core subunits, reduced *F*_v_/*F*_m_ value, and sensitivity to high light intensities (Wei *et al.*, 2010). Moreover, the Arabidopsis *pam68* mutant with impaired D1 processing is most affected in the assembly of PSII dimers and supercomplexes (Armbruster *et al.*, 2010), just like the *Chlamydomonas lpa2* mutant.

### Side effects of impaired PSII assembly in the *lpa2* mutant

While levels of the cytochrome *b*_6_*f* complex and of PSI are unaltered in the *lpa2* mutant compared with the wild type, levels of the ATP synthase are ~29% higher and levels of LHCII ~27% higher in the mutant (Table 1). Possibly, the large pools of ‘free’ LHCII and partially assembled PSII in the form of the RC47 complex are the reason for the increased high light sensitivity of the *lpa2* mutant compared with the wild type (Fig. 1F). The imbalances in major thylakoid membrane complexes might also be the cause of the aberrant structures we observed in thylakoid membrane stacks in the mutant (Fig. 2).

We also observed a more disconnected LHCI antenna in the mutant compared with the wild type (Figs 4A, 5), possibly because the mutant reduces excitation energy to PSI as a regulative adaptation to the low amounts of PSII. The disconnected LHCI antenna proteins displayed characteristic migration profiles pointing to the disconnection of a major LHCI complex composed of LHCA1,3–8 (d in Fig. 4A) and a minor one composed of LHCA2,9 (g in Fig. 4A). The prominent LHCA1,3–8 complex appeared to disassemble into LHCA1,4–6, LHCA4–6, and LHCA1,7,8 subcomplexes (e–g in Fig. 4A). This is in perfect agreement with a model proposed recently in which LHCA1,3–8 bind at one side of the PSI core in two layers, with LHCA1,3,7,8 forming the inner and LHCA1,4–6 the outer layer; LHCA2,9 bind at the opposite side (Ozawa *et al.*, 2018). A major PSI complex accumulating in the mutant, and to a lesser extent also in the wild type, specifically lacks LHCA2,9, PSAG, and PSAH (a in Fig. 4A). Again, this is in perfect agreement with the model that LHCA2,9 are flanked by PSAG and PSAH (Ozawa *et al.*, 2018) and indicates that the uncoupling of these proteins is the prime response in the *lpa2* mutant to reduce the PSI antenna size. Nevertheless, a minor PSI complex contains LHCA2,9 but lacks LHCA1,3–8 (b in Fig. 4A), indicating that the disconnection of LHCA2,9 must not necessarily precede the disconnection of LHCA1,3–8. Smaller PSI complexes, presumably lacking LHCA1,3–8, also lack PSAK (c in Fig. 4A), again in line with the proposed role of this subunit in flanking LHCA1,3–8 (Ozawa *et al.*, 2018).

### Novel proteins that potentially interact with PSII

Complexome profiling allowed us to identify three novel proteins potentially interacting with PSII. One of them, PBA1, co-migrated with PSII subcomplexes. The distinct migration profiles of PSII subcomplexes in wild type and *lpa2* mutant increased the confidence of true co-migration with PBA1, and the detection of two peptides the confidence of its identification (Figs 4A, 6; Supplementary Dataset S3). PBA1 is conserved only in green algae, diatoms, Eustigmatophytes, and brown algae (Fig. 7A), which might explain why it has not been detected yet. Its small size (6.4 kDa) and the predicted transmembrane helix are typical features of small PSII core subunits (Pagliano *et al.*, 2013).

Like PBA1, CGLD16 is a small protein of 7.9 kDa with a predicted transmembrane helix. It co-migrated only with PSII monomers and RC47 and, as expected, more with monomers in the wild type, and more with RC47 in the *lpa2* mutant (Figs 4A, 6). Although its detection was based on a single peptide, this was with a high identification score (Supplementary Dataset S3). This protein is conserved in the green lineage and diatoms (Fig. 7B) and could potentially represent a novel factor involved in PSII assembly, repair, or the regulation of PSII complex dynamics.

The small size of PBA1 and CGLD16 justified that their potential interaction with the different PSII assemblies did not change their migration properties. This is different for the third protein found to potentially interact exclusively with RC47 in the *lpa2* mutant: PAO5. PAO5 has a predicted molecular mass of 52 kDa and was identified with three peptides of low ion intensity (Supplementary Dataset S3). Its co-migration with RC47 implies that PAO5 has replaced another protein of similar molecular mass in RC47, such as CP47, D1, or D2. Therefore, we cannot exclude that PAO5 interacts with another protein complex present only in the *lpa2* mutant and just by chance co-migrates with RC47. PAO5 encodes a protein with Rieske-type [2Fe–2S] domain and a pheophorbide *a* oxygenase domain. It is in the same gene family as the Arabidopsis ACCELERATED CELL DEATH 1 (ACD1) protein that is involved in chlorophyll *a* breakdown to avoid chlorophyll phototoxicity (Pruzinská *et al.*, 2003; Kuai *et al.*, 2018). However, the closest homologs of PAO5 are cyanobacterial proteins, while close PAO5 homologs in Arabidopsis are missing (Fig. 7C; Supplementary Fig. S5). Given the 6-fold higher abundance of RC47 in the mutant compared with wild type and the necessity to cope with chlorophyll phototoxicity upon degradation of non-productive PSII assembly intermediates, we believe that the identification of PAO5 only in the *lpa2* mutant is interesting. Clearly, its direct interaction partners need to be identified by co-immunoprecipitation or related approaches.

In this study, we analysed our complexome profiling dataset with a focus on the major photosynthetic complexes in the thylakoid membranes. However, the dataset is so rich that proteins involved in many other aspects can be investigated, such as complexes of the mitochondrial respiration chain or complexes formed by TCA cycle enzymes (Rugen *et al.*, 2021). These are in the dataset because tubular mitochondria are closely connected with chloroplasts in *Chlamydomonas* (Schötz *et al.*, 1972) and contaminate thylakoid preparations.

## Supplementary data

The following supplementary data are available at *JXB* online.

Fig. S1. Analysis of the CIB1 integration site by PCR.

Fig. S2. Light microscopy images of wild type and *lpa2* mutant cells.

Fig. S3. Screening of transformants expressing LPA2 with (lpa2-cHA) or without (lpa2-c) a 3×HA tag.

Fig. S4. BN-PAGE for complexome profiling.

Fig. S5. Phylogenetic tree of pheophorbide *a* oxygenases.

Table S1. Proteins involved in PSII assembly, repair, or complex dynamics that have clear homologs in *Chlamydomonas* and are present in the complexome profiling dataset.

Dataset S1. List of all 1734 proteins identified by complexome profiling and links to migration profiles.

Dataset S2. Heat maps of migration profiles of all identified proteins of wild type and *lpa2* mutant.

Dataset S3. Identified peptides of selected proteins with ID scores and ion intensities.

erab390_suppl_Supplementary_Dataset_S1

erab390_suppl_Supplementary_Dataset_S2

erab390_suppl_Supplementary_Dataset_S3

erab390_suppl_Supplementary_Table_S1_Figures_S1-S5

## Data Availability

All data supporting the findings of this study are available within the paper and within its supplementary data published online.
