# Supplementary material for: Complexome profiling on the Chlamydomonas lpa2 mutant reveals insights into PSII biogenesis and new PSII associated proteins
Source: J Exp Bot. 2021 Aug 26;73(1):245–62. doi: 10.1093/jxb/erab390 (PMC8730698; doi:10.1093/jxb/erab390)
Supplement: erab390_suppl_Supplementary_Dataset_S1 [file erab390_suppl_supplementary_dataset_s1.zip › Supplemental Dataset 1 - Excel List and all profiles/plots/AAA1_Cre08.g358526.html]

### 

Trivial name: AAA1  
  
Euclidean distance: 569492.10  
Mean Intensity (WT): 57147.60  
Mean Intensity (Mut): 70571.45  
Distance: 8.07  
  
MapMan: transport.misc;transport.metabolite transporters at the mitochondrial membrane;transport.metabolite transporters at the envelope membrane  
  
p value of intensity sums Welch test: 0.4065
