# Supplementary material for: Complexome profiling on the Chlamydomonas lpa2 mutant reveals insights into PSII biogenesis and new PSII associated proteins
Source: J Exp Bot. 2021 Aug 26;73(1):245–62. doi: 10.1093/jxb/erab390 (PMC8730698; doi:10.1093/jxb/erab390)
Supplement: erab390_suppl_Supplementary_Dataset_S1 [file erab390_suppl_supplementary_dataset_s1.zip › Supplemental Dataset 1 - Excel List and all profiles/plots/AAT1_Cre10.g451950.html]

### 

Trivial name: AAT1  
  
Euclidean distance: 92144.30  
Mean Intensity (WT): 14164.62  
Mean Intensity (Mut): 11730.41  
Distance: 6.51  
  
MapMan: amino acid metabolism.synthesis.serine-glycine-cysteine group.glycine.glycine transaminase;amino acid metabolism.synthesis.central amino acid metabolism.alanine.alanine aminotransferase;PS.photorespiration.aminotransferases peroxisomal  
  
p value of intensity sums Welch test: 0.7475
