# Supplementary material for: Complexome profiling on the Chlamydomonas lpa2 mutant reveals insights into PSII biogenesis and new PSII associated proteins
Source: J Exp Bot. 2021 Aug 26;73(1):245–62. doi: 10.1093/jxb/erab390 (PMC8730698; doi:10.1093/jxb/erab390)
Supplement: erab390_suppl_Supplementary_Dataset_S1 [file erab390_suppl_supplementary_dataset_s1.zip › Supplemental Dataset 1 - Excel List and all profiles/plots/ACC1_Cre12.g519100.html]

### 

Trivial name: ACC1  
  
Euclidean distance: 26873.30  
Mean Intensity (WT): 5169.67  
Mean Intensity (Mut): 3576.51  
Distance: 5.20  
  
MapMan: secondary metabolism.unspecified;lipid metabolism.FA synthesis and FA elongation.acetyl CoA carboxylation.heteromeric complex.alpha carboxyltransferase  
  
p value of intensity sums Welch test: 0.4412
