# Supplementary material for: Complexome profiling on the Chlamydomonas lpa2 mutant reveals insights into PSII biogenesis and new PSII associated proteins
Source: J Exp Bot. 2021 Aug 26;73(1):245–62. doi: 10.1093/jxb/erab390 (PMC8730698; doi:10.1093/jxb/erab390)
Supplement: erab390_suppl_Supplementary_Dataset_S1 [file erab390_suppl_supplementary_dataset_s1.zip › Supplemental Dataset 1 - Excel List and all profiles/plots/ACS3_Cre07.g353450.html]

### 

Trivial name: ACS3  
  
Euclidean distance: 394711.31  
Mean Intensity (WT): 31612.05  
Mean Intensity (Mut): 47118.39  
Distance: 8.38  
  
MapMan: major CHO metabolism.degradation.sucrose.invertases;secondary metabolism.phenylpropanoids.lignin biosynthesis.4CL;lipid metabolism.FA synthesis and FA elongation.acyl CoA ligase  
  
p value of intensity sums Welch test: 0.2341
