# Supplementary material for: Complexome profiling on the Chlamydomonas lpa2 mutant reveals insights into PSII biogenesis and new PSII associated proteins
Source: J Exp Bot. 2021 Aug 26;73(1):245–62. doi: 10.1093/jxb/erab390 (PMC8730698; doi:10.1093/jxb/erab390)
Supplement: erab390_suppl_Supplementary_Dataset_S1 [file erab390_suppl_supplementary_dataset_s1.zip › Supplemental Dataset 1 - Excel List and all profiles/plots/AGS1_Cre09.g416050.html]

### 

Trivial name: AGS1  
  
Euclidean distance: 3157.47  
Mean Intensity (WT): 205.51  
Mean Intensity (Mut): 85.66  
Distance: 15.36  
  
MapMan: amino acid metabolism.synthesis.glutamate family.arginine.argininosuccinate synthase;amino acid metabolism.synthesis.glutamate family.arginine  
  
p value of intensity sums Welch test: 0.5694
