# Supplementary material for: Complexome profiling on the Chlamydomonas lpa2 mutant reveals insights into PSII biogenesis and new PSII associated proteins
Source: J Exp Bot. 2021 Aug 26;73(1):245–62. doi: 10.1093/jxb/erab390 (PMC8730698; doi:10.1093/jxb/erab390)
Supplement: erab390_suppl_Supplementary_Dataset_S1 [file erab390_suppl_supplementary_dataset_s1.zip › Supplemental Dataset 1 - Excel List and all profiles/plots/AGT1_Cre06.g294650.html]

### 

Trivial name: AGT1  
  
Euclidean distance: 177477.96  
Mean Intensity (WT): 23061.78  
Mean Intensity (Mut): 16652.54  
Distance: 7.70  
  
MapMan: amino acid metabolism.synthesis.serine-glycine-cysteine group.glycine.serine glyoxylate aminotransferase;amino acid metabolism.synthesis.central amino acid metabolism.alanine.alanine-glyoxylate aminotransferase;PS.photorespiration.aminotransferases peroxisomal  
  
p value of intensity sums Welch test: 0.5293
