# Supplementary material for: Complexome profiling on the Chlamydomonas lpa2 mutant reveals insights into PSII biogenesis and new PSII associated proteins
Source: J Exp Bot. 2021 Aug 26;73(1):245–62. doi: 10.1093/jxb/erab390 (PMC8730698; doi:10.1093/jxb/erab390)
Supplement: erab390_suppl_Supplementary_Dataset_S1 [file erab390_suppl_supplementary_dataset_s1.zip › Supplemental Dataset 1 - Excel List and all profiles/plots/AMA2_Cre08.g362450.html]

### 

Trivial name: AMA2  
  
Euclidean distance: 45100.76  
Mean Intensity (WT): 1411.01  
Mean Intensity (Mut): 440.54  
Distance: 31.96  
  
MapMan: major CHO metabolism.degradation.starch.starch cleavage.alpha amylase;major CHO metabolism.degradation.starch.starch cleavage  
  
p value of intensity sums Welch test: 0.5643
