# Supplementary material for: Complexome profiling on the Chlamydomonas lpa2 mutant reveals insights into PSII biogenesis and new PSII associated proteins
Source: J Exp Bot. 2021 Aug 26;73(1):245–62. doi: 10.1093/jxb/erab390 (PMC8730698; doi:10.1093/jxb/erab390)
Supplement: erab390_suppl_Supplementary_Dataset_S1 [file erab390_suppl_supplementary_dataset_s1.zip › Supplemental Dataset 1 - Excel List and all profiles/plots/APX4_Cre05.g233900.html]

### 

Trivial name: APX4  
  
Euclidean distance: 187270.72  
Mean Intensity (WT): 11670.74  
Mean Intensity (Mut): 17646.62  
Distance: 10.61  
  
MapMan: redox.ascorbate and glutathione.ascorbate  
  
p value of intensity sums Welch test: 0.0715
