# Supplementary material for: Complexome profiling on the Chlamydomonas lpa2 mutant reveals insights into PSII biogenesis and new PSII associated proteins
Source: J Exp Bot. 2021 Aug 26;73(1):245–62. doi: 10.1093/jxb/erab390 (PMC8730698; doi:10.1093/jxb/erab390)
Supplement: erab390_suppl_Supplementary_Dataset_S1 [file erab390_suppl_supplementary_dataset_s1.zip › Supplemental Dataset 1 - Excel List and all profiles/plots/ASN1_Cre01.g004300.html]

### 

Trivial name: ASN1  
  
Euclidean distance: 122218.60  
Mean Intensity (WT): 2198.07  
Mean Intensity (Mut): 7870.20  
Distance: 15.53  
  
MapMan: amino acid metabolism.synthesis.aspartate family.asparagine.asparagine synthetase;amino acid metabolism.synthesis.aspartate family.asparagine  
  
p value of intensity sums Welch test: 0.3079
