# Supplementary material for: Complexome profiling on the Chlamydomonas lpa2 mutant reveals insights into PSII biogenesis and new PSII associated proteins
Source: J Exp Bot. 2021 Aug 26;73(1):245–62. doi: 10.1093/jxb/erab390 (PMC8730698; doi:10.1093/jxb/erab390)
Supplement: erab390_suppl_Supplementary_Dataset_S1 [file erab390_suppl_supplementary_dataset_s1.zip › Supplemental Dataset 1 - Excel List and all profiles/plots/AST3_Cre02.g097900.html]

### 

Trivial name: AST3  
  
Euclidean distance: 287260.39  
Mean Intensity (WT): 1109.26  
Mean Intensity (Mut): 11950.95  
Distance: 24.04  
  
MapMan: amino acid metabolism.synthesis.central amino acid metabolism.aspartate.aspartate aminotransferase  
  
p value of intensity sums Welch test: 0.4353
