# Supplementary material for: Complexome profiling on the Chlamydomonas lpa2 mutant reveals insights into PSII biogenesis and new PSII associated proteins
Source: J Exp Bot. 2021 Aug 26;73(1):245–62. doi: 10.1093/jxb/erab390 (PMC8730698; doi:10.1093/jxb/erab390)
Supplement: erab390_suppl_Supplementary_Dataset_S1 [file erab390_suppl_supplementary_dataset_s1.zip › Supplemental Dataset 1 - Excel List and all profiles/plots/ATO1_Cre17.g723650.html]

### 

Trivial name: ATO1  
  
Euclidean distance: 22292.42  
Mean Intensity (WT): 2530.69  
Mean Intensity (Mut): 2788.23  
Distance: 8.00  
  
MapMan: amino acid metabolism.degradation.branched chain group.shared  
  
p value of intensity sums Welch test: 0.8522
