# Supplementary material for: Complexome profiling on the Chlamydomonas lpa2 mutant reveals insights into PSII biogenesis and new PSII associated proteins
Source: J Exp Bot. 2021 Aug 26;73(1):245–62. doi: 10.1093/jxb/erab390 (PMC8730698; doi:10.1093/jxb/erab390)
Supplement: erab390_suppl_Supplementary_Dataset_S1 [file erab390_suppl_supplementary_dataset_s1.zip › Supplemental Dataset 1 - Excel List and all profiles/plots/ATP2_Cre17.g698000.html]

### 

Trivial name: ATP2  
  
Euclidean distance: 1914910.63  
Mean Intensity (WT): 830726.66  
Mean Intensity (Mut): 829310.10  
Distance: 2.31  
  
MapMan: mitochondrial electron transport / ATP synthesis.F1-ATPase  
  
p value of intensity sums Welch test: 0.9934
