# Supplementary material for: Complexome profiling on the Chlamydomonas lpa2 mutant reveals insights into PSII biogenesis and new PSII associated proteins
Source: J Exp Bot. 2021 Aug 26;73(1):245–62. doi: 10.1093/jxb/erab390 (PMC8730698; doi:10.1093/jxb/erab390)
Supplement: erab390_suppl_Supplementary_Dataset_S1 [file erab390_suppl_supplementary_dataset_s1.zip › Supplemental Dataset 1 - Excel List and all profiles/plots/ATP6_Cre01.g018800.html]

### 

Trivial name: ATP6  
  
Euclidean distance: 56737.06  
Mean Intensity (WT): 21354.56  
Mean Intensity (Mut): 19534.97  
Distance: 2.66  
  
MapMan: mitochondrial electron transport / ATP synthesis.F1-ATPase;transport.p- and v-ATPases.H+-transporting two-sector ATPase  
  
p value of intensity sums Welch test: 0.5010
