# Supplementary material for: Complexome profiling on the Chlamydomonas lpa2 mutant reveals insights into PSII biogenesis and new PSII associated proteins
Source: J Exp Bot. 2021 Aug 26;73(1):245–62. doi: 10.1093/jxb/erab390 (PMC8730698; doi:10.1093/jxb/erab390)
Supplement: erab390_suppl_Supplementary_Dataset_S1 [file erab390_suppl_supplementary_dataset_s1.zip › Supplemental Dataset 1 - Excel List and all profiles/plots/ATPD_Cre11.g467569.html]

### 

Trivial name: ATPD  
  
Euclidean distance: 397362.82  
Mean Intensity (WT): 150000.81  
Mean Intensity (Mut): 161736.66  
Distance: 2.46  
  
MapMan: PS.lightreaction.ATP synthase.delta chain;PS.lightreaction.ATP synthase  
  
p value of intensity sums Welch test: 0.3925
