# Supplementary material for: Complexome profiling on the Chlamydomonas lpa2 mutant reveals insights into PSII biogenesis and new PSII associated proteins
Source: J Exp Bot. 2021 Aug 26;73(1):245–62. doi: 10.1093/jxb/erab390 (PMC8730698; doi:10.1093/jxb/erab390)
Supplement: erab390_suppl_Supplementary_Dataset_S1 [file erab390_suppl_supplementary_dataset_s1.zip › Supplemental Dataset 1 - Excel List and all profiles/plots/atpH_Cre-1.g2717044.html]

### 

Trivial name: atpH  
  
Euclidean distance: 19020.90  
Mean Intensity (WT): 335.61  
Mean Intensity (Mut): 699.80  
Distance: 27.18  
  
MapMan:   
  
p value of intensity sums Welch test: 0.6613
