# Supplementary material for: Complexome profiling on the Chlamydomonas lpa2 mutant reveals insights into PSII biogenesis and new PSII associated proteins
Source: J Exp Bot. 2021 Aug 26;73(1):245–62. doi: 10.1093/jxb/erab390 (PMC8730698; doi:10.1093/jxb/erab390)
Supplement: erab390_suppl_Supplementary_Dataset_S1 [file erab390_suppl_supplementary_dataset_s1.zip › Supplemental Dataset 1 - Excel List and all profiles/plots/ATPVA1_Cre10.g461050.html]

### 

Trivial name: ATPVA1  
  
Euclidean distance: 40970.84  
Mean Intensity (WT): 3369.76  
Mean Intensity (Mut): 4080.29  
Distance: 10.04  
  
MapMan: transport.p- and v-ATPases.H+-transporting two-sector ATPase;transport.p- and v-ATPases  
  
p value of intensity sums Welch test: 0.4958
