# Supplementary material for: Complexome profiling on the Chlamydomonas lpa2 mutant reveals insights into PSII biogenesis and new PSII associated proteins
Source: J Exp Bot. 2021 Aug 26;73(1):245–62. doi: 10.1093/jxb/erab390 (PMC8730698; doi:10.1093/jxb/erab390)
Supplement: erab390_suppl_Supplementary_Dataset_S1 [file erab390_suppl_supplementary_dataset_s1.zip › Supplemental Dataset 1 - Excel List and all profiles/plots/ATPVL1_Cre06.g257450.html]

### 

Trivial name: ATPVL1  
  
Euclidean distance: 20763.99  
Mean Intensity (WT): 1667.23  
Mean Intensity (Mut): 2552.78  
Distance: 8.13  
  
MapMan: transport.p- and v-ATPases.H+-transporting two-sector ATPase.subunit C;transport.p- and v-ATPases.H+-transporting two-sector ATPase  
  
p value of intensity sums Welch test: 0.1524
