# Supplementary material for: Complexome profiling on the Chlamydomonas lpa2 mutant reveals insights into PSII biogenesis and new PSII associated proteins
Source: J Exp Bot. 2021 Aug 26;73(1):245–62. doi: 10.1093/jxb/erab390 (PMC8730698; doi:10.1093/jxb/erab390)
Supplement: erab390_suppl_Supplementary_Dataset_S1 [file erab390_suppl_supplementary_dataset_s1.zip › Supplemental Dataset 1 - Excel List and all profiles/plots/BCA2_Cre05.g245900.html]

### 

Trivial name: BCA2  
  
Euclidean distance: 5787.15  
Mean Intensity (WT): 438.03  
Mean Intensity (Mut): 509.52  
Distance: 11.36  
  
MapMan: misc.aminotransferases.aminotransferase class IV family protein;Co-factor and vitamine metabolism.pantothenate.branched-chain amino acid aminotransferase;secondary metabolism.sulfur-containing.glucosinolates.synthesis.aliphatic.branched-chain amino acid aminotransferase (BCAT/MAAT);amino acid metabolism.synthesis.branched chain group.common.branched-chain-amino-acid aminotransferase  
  
p value of intensity sums Welch test: 0.8626
