# Supplementary material for: Complexome profiling on the Chlamydomonas lpa2 mutant reveals insights into PSII biogenesis and new PSII associated proteins
Source: J Exp Bot. 2021 Aug 26;73(1):245–62. doi: 10.1093/jxb/erab390 (PMC8730698; doi:10.1093/jxb/erab390)
Supplement: erab390_suppl_Supplementary_Dataset_S1 [file erab390_suppl_supplementary_dataset_s1.zip › Supplemental Dataset 1 - Excel List and all profiles/plots/BCC1_Cre17.g715250.html]

### 

Trivial name: BCC1  
  
Euclidean distance: 5349.70  
Mean Intensity (WT): 147.77  
Mean Intensity (Mut): 240.17  
Distance: 22.27  
  
MapMan: Co-factor and vitamine metabolism.biotin;lipid metabolism.FA synthesis and FA elongation.acetyl CoA carboxylation.heteromeric complex.biotin carboxyl carrier protein;lipid metabolism.FA synthesis and FA elongation.acetyl CoA carboxylation  
  
p value of intensity sums Welch test: 0.5503
