# Supplementary material for: Complexome profiling on the Chlamydomonas lpa2 mutant reveals insights into PSII biogenesis and new PSII associated proteins
Source: J Exp Bot. 2021 Aug 26;73(1):245–62. doi: 10.1093/jxb/erab390 (PMC8730698; doi:10.1093/jxb/erab390)
Supplement: erab390_suppl_Supplementary_Dataset_S1 [file erab390_suppl_supplementary_dataset_s1.zip › Supplemental Dataset 1 - Excel List and all profiles/plots/CAG3_Cre09.g415850.html]

### 

Trivial name: CAG3  
  
Euclidean distance: 184618.36  
Mean Intensity (WT): 21148.98  
Mean Intensity (Mut): 18526.48  
Distance: 8.73  
  
MapMan: mitochondrial electron transport / ATP synthesis.NADH-DH (type I).complex I.carbonic anhydrase  
  
p value of intensity sums Welch test: 0.5618
