# Supplementary material for: Complexome profiling on the Chlamydomonas lpa2 mutant reveals insights into PSII biogenesis and new PSII associated proteins
Source: J Exp Bot. 2021 Aug 26;73(1):245–62. doi: 10.1093/jxb/erab390 (PMC8730698; doi:10.1093/jxb/erab390)
Supplement: erab390_suppl_Supplementary_Dataset_S1 [file erab390_suppl_supplementary_dataset_s1.zip › Supplemental Dataset 1 - Excel List and all profiles/plots/CARB1_Cre08.g359350.html]

### 

Trivial name: CARB1  
  
Euclidean distance: 1468.61  
Mean Intensity (WT): 70.29  
Mean Intensity (Mut): 37.49  
Distance: 20.89  
  
MapMan: amino acid metabolism.degradation.branched chain group.leucine;lipid metabolism.FA synthesis and FA elongation.acetyl CoA carboxylation.heteromeric complex.biotin carboxylase;lipid metabolism.FA synthesis and FA elongation.acetyl CoA carboxylation  
  
p value of intensity sums Welch test: 0.7077
