# Supplementary material for: Complexome profiling on the Chlamydomonas lpa2 mutant reveals insights into PSII biogenesis and new PSII associated proteins
Source: J Exp Bot. 2021 Aug 26;73(1):245–62. doi: 10.1093/jxb/erab390 (PMC8730698; doi:10.1093/jxb/erab390)
Supplement: erab390_suppl_Supplementary_Dataset_S1 [file erab390_suppl_supplementary_dataset_s1.zip › Supplemental Dataset 1 - Excel List and all profiles/plots/CCB4_Cre08.g382300.html]

### 

Trivial name: CCB4  
  
Euclidean distance: 9521.20  
Mean Intensity (WT): 550.65  
Mean Intensity (Mut): 443.76  
Distance: 17.29  
  
MapMan: protein.assembly and cofactor ligation  
  
p value of intensity sums Welch test: 0.3180
