# Supplementary material for: Complexome profiling on the Chlamydomonas lpa2 mutant reveals insights into PSII biogenesis and new PSII associated proteins
Source: J Exp Bot. 2021 Aug 26;73(1):245–62. doi: 10.1093/jxb/erab390 (PMC8730698; doi:10.1093/jxb/erab390)
Supplement: erab390_suppl_Supplementary_Dataset_S1 [file erab390_suppl_supplementary_dataset_s1.zip › Supplemental Dataset 1 - Excel List and all profiles/plots/CCS1_Cre13.g575000.html]

### 

Trivial name: CCS1  
  
Euclidean distance: 7381.64  
Mean Intensity (WT): 1350.15  
Mean Intensity (Mut): 1103.30  
Distance: 5.47  
  
MapMan: mitochondrial electron transport / ATP synthesis.cytochrome c  
  
p value of intensity sums Welch test: 0.1389
