# Supplementary material for: Complexome profiling on the Chlamydomonas lpa2 mutant reveals insights into PSII biogenesis and new PSII associated proteins
Source: J Exp Bot. 2021 Aug 26;73(1):245–62. doi: 10.1093/jxb/erab390 (PMC8730698; doi:10.1093/jxb/erab390)
Supplement: erab390_suppl_Supplementary_Dataset_S1 [file erab390_suppl_supplementary_dataset_s1.zip › Supplemental Dataset 1 - Excel List and all profiles/plots/CGLD27_Cre05.g237050.html]

### 

Trivial name: CGLD27  
  
Euclidean distance: 5624.59  
Mean Intensity (WT): 113.48  
Mean Intensity (Mut): 307.17  
Distance: 18.31  
  
MapMan:   
  
p value of intensity sums Welch test: 0.2041
