# Supplementary material for: Complexome profiling on the Chlamydomonas lpa2 mutant reveals insights into PSII biogenesis and new PSII associated proteins
Source: J Exp Bot. 2021 Aug 26;73(1):245–62. doi: 10.1093/jxb/erab390 (PMC8730698; doi:10.1093/jxb/erab390)
Supplement: erab390_suppl_Supplementary_Dataset_S1 [file erab390_suppl_supplementary_dataset_s1.zip › Supplemental Dataset 1 - Excel List and all profiles/plots/CGLD38_Cre13.g562750.html]

### 

Trivial name: CGLD38  
  
Euclidean distance: 2123.60  
Mean Intensity (WT): 231.42  
Mean Intensity (Mut): 152.26  
Distance: 9.18  
  
MapMan:   
  
p value of intensity sums Welch test: 0.4630
