# Supplementary material for: Complexome profiling on the Chlamydomonas lpa2 mutant reveals insights into PSII biogenesis and new PSII associated proteins
Source: J Exp Bot. 2021 Aug 26;73(1):245–62. doi: 10.1093/jxb/erab390 (PMC8730698; doi:10.1093/jxb/erab390)
Supplement: erab390_suppl_Supplementary_Table_S1_Figures_S1-S5 [file erab390_suppl_supplementary_table_s1_figures_s1-s5.pdf]

**SUPPLEMENTARY INFORMATION**

**Title:** Complexome profiling on the *Chlamydomonas lpa2* mutant reveals insights into PSII biogenesis and new PSII associated proteins

**Authors:** Benjamin Spaniol[1,§], Julia Lang[1,$], Benedikt Venn[2,§], Lara Schake[1], Frederik Sommer[1], Matthieu Mustas[3], Stefan Geimer[4], Francis-André Wollman[3], Yves Choquet[3], Timo Mühlhaus[2], and Michael Schroda[1, ¶]

**Affiliations:**

[1] Molekulare Biotechnologie & Systembiologie, TU Kaiserslautern, Paul-Ehrlich Straße 23, D-67663 Kaiserslautern, Germany

[2] Computational Systems Biology, TU Kaiserslautern, Paul-Ehrlich Straße 23, D-67663 Kaiserslautern, Germany

[3] Biologie du Chloroplaste et Perception de la Lumière chez les Microalgues, Institut de Biologie Physico-Chimique, UMR CNRS/UPMC 7141, Paris, France

[4] Zellbiologie/Elektronenmikroskopie, Universität Bayreuth, 95440 Bayreuth, Germany

[§] These authors contributed equally to this work

[§] These authors contributed equally to this work

[¶] Corresponding author : Michael Schroda (schroda@bio.uni-kl.de)

**Supplementary Table S1.** Proteins involved in PSII assembly, repair, or complex dynamics that have clear homologs in *Chlamydomonas* and are present in the complexome profiling dataset. The data are from Lu (2016). Ath – *Arabidopsis thaliana*; Syn – *Synechocystis*; Cre – *Chlamydomonas reinhardtii*.

| Locus Ath | Name Ath/ Syn | Name Cre | Locus Cre | Description from Lu (2016) for Ath homolog |
|---|---|---|---|---|
| At2g47450 | cpSRP43 | SRP43 | Cre04.g231026 | Insertion and assembly of PSII proteins such as D1, D2, and CP47, and LHCII subunits |
| At2g28800 | ALB3 | ALB3.1 | Cre06.g251900 | Insertion and assembly of PSII proteins such as D1, D2, and CP47, and LHCII subunits |
| | | ALB3.2 | Cre17.g729800 | |
| At2g18710 | cpSecY1 | SECY1 | Cre16.g681900 | Insertion and assembly of PSII proteins such as PsbO |
| At5g28750 | Tha4 | TATA | Cre10.g438550 | Insertion and assembly of PSII proteins such as PsbP and PsbQ |
| At5g52440 | HCF106 | TATB | Cre08.g371650 | Insertion and assembly of PSII proteins such as PsbP and PsbQ |
| At2g01110 | cpTatC | TATC | Cre03.g145887 | Insertion and assembly of PSII proteins such as PsbP and PsbQ |
| At1g05810 | cpRabA5e | RAB11 | Cre03.g189250 | Transport of PSII proteins such as LHCB1, LHCB3 and CP47 to and from thylakoids |
| At2g20890 | THF1/PSB29 | THF1, PSB29 | Cre13.g562850 | Dynamics of PSII-LHCII supercomplexes |
| At5g12130 | TERC | TERC | Cre17.g712400 | Co-translational insertion of PSII proteins such as D1, D2, and CP43 |
| At1g65260 | VIPP1 | VIPP1 | Cre13.g583550 | Lipid microdomain |
| At3g22840 | ELIP1 | ELIP1 | Cre16.g679250 | Binding of chlorophyll and/or stability of pigment-binding proteins and complexes during photoinhibition? |
| At4g14690 | ELIP2 | ELIP2 | Cre09.g393173 | Binding of chlorophyll and/or stability of pigment-binding proteins and complexes during photoinhibition? |
| At4g17600 | SEP3.1/LIL3.1 | LHL3, LIL3 | Cre03.g199535 | Anchoring geranylgeranyl reductase to thylakoid membranes; stabilizing LHCII |
| At1g02910 | LPA1/PratA | REP27, LPA1 | Cre10.g430150 | Biogenesis and assembly of the D1 protein |
| At1g55480 | MET1 | TEF30, MET1 | Cre01.g031100 | Supercomplex formation in PSII repair |
| At1g75690 | LQY1 | ZNJ1, LQY1 | Cre11.g475850 | Disassembly, folding, and/or reassembly of cysteine-containing PSII subunits and complexes and/or D1 synthesis and turnover during PSII repair |
| At1g77510 | PDI6/PDIL1-2 | RB60 | Cre02.g088200 | Regulation of D1 synthesis |
| At4g35760 | LTO1 | CPLD41, VKE1, LTO1 | Cre12.g493150 | Disulfide bond formation in PsbO |
| At1g54500 | RBD1 | RBD1 | Cre07.g315150 | PSII assembly and stability |
| At3g01480 | CYP38/TLP40 | CYN38, TLP40 | Cre03.g189800 | PPIase; Inhibiting dephosphorylation of PSII subunits during PSII repair; conversion of PSII core monomers to PSII supercomplexes |
| At3g60370 | FKBP20-2 | FKB20 | Cre13.g577850 | PPIase; Formation of PSII-LHCII supercomplexes under normal and high light |
| At5g01920 | STN8 | STN7 | Cre12.g483650 | Phosphorylation of D1, D2, CP43, and PsbH |
| At1g54780 | TLP18.3 | TEF8, TLP18.3 | Cre03.g182150 | D1 degradation and PSII dimerization; dephosphorylation of PSII core proteins (e.g.,D1 and D2) |
| At1g50250 | FtsH1 | FTSH1 | Cre12.g485800 | Degradation of photodamaged D1 |
| At2g30950 | FtsH2/VAR2 | FTSH2 | Cre17.g720050 | Chloroplast biogenesis; thylakoid formation; degradation of photodamaged D1 |
| At3g27925 | Deg1 | DEG1B | Cre14.g630550 | Degradation of plastocyanin and PsbO, and photodamaged D1; integration of newly synthesized PSII subunits such as D1, D2, CP43, and CP47, into PSII complexes |
| | | DEG1C | Cre12.g498500 | Stress-induced degradation of LHCB6; a minor protease in in vivo degradation of photodamaged D1 |
| At1g03600 | PSB27 | CPLD45, PSB27 | Cre05.g243800 | C-terminal processing of D1 during PSII repair? |
| At1g05385 | PSB27-H2/LPA19 | CGL54, LPA19 | Cre02.g073850 | C-terminal processing during de novo PSII assembly |
| At5g23120 | HCF136 (YCF48) | HCF136, YCF48 | Cre06.g273700 | Assembly of PSII reaction-center complexes such as RC, RC47a, and RC47b |
| AtCg00700 | PsbN/PBF1 | PsbN | cp-encoded | Assembly of PSII minimal reaction-center complexes; regulation of PSII core and antenna protein phosphorylation |
| At4g28660 | PSB28 | PSB28 | Cre10.g440450 | Biogenesis of chlorophyll-binding proteins such as CP47, PsaA, and PsaB |
| At5g51545 | LPA2 | LPA2 | Cre02.g105650 | Synthesis and assembly of CP43 |
| At1g71500 | PSB33 | TEF5, PSB33 | Cre09.g411200 | Association of LHCII with PSII |

## SUPPLEMENTARY FIGURES AND FIGURE LEGENDS

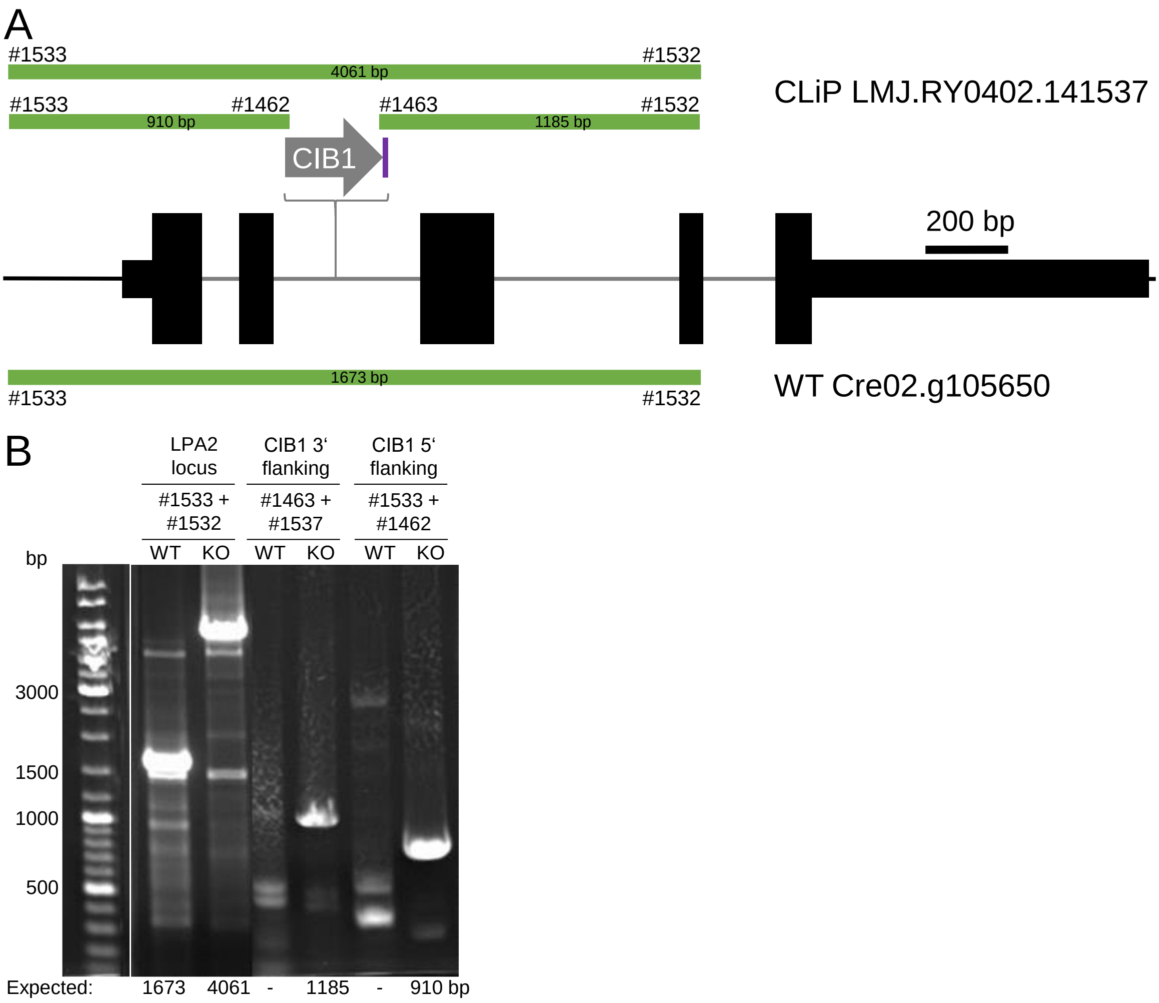


C

| Primer # | Name | Sequence |
|---|---|---|
| 1462 | OMJ 913 | GCACCAATCATGTCAAGCCT |
| 1463 | OMJ 944 | GACGTTACAGCACACCCTTG |
| 1533 | LPA2_for | TGTGGAGCTTCTGCATTGTC |
| 1532 | LPA2_rev | TTGGGGTAGAGGTTGTCCTG |

**Supplementary Fig. S1.** Analysis of the CIB1 integration site by PCR. (A) Gene model of the *LPA2* gene with exons shown as black boxes and introns as thin grey lines. The integration site of the CIB1 cassette in the second intron is shown. The purple box indicates a 165-bp fragment derived from the 19$^{th}$ intron of gene Cre13.g573450 in reverse orientation that has integrated together with the CIB1 cassette. Green bars indicate the expected PCR products on mutant DNA (CLiP, top) and wild-type DNA (WT, bottom). Numbers in the bars give their sizes and flanking numbers the primer number as provided in the table. (B) PCR products on genomic DNA from wild type (WT) and the *lpa2* mutant from the CLiP collection (KO) were separated on an agarose gel and stained with Gel Red. Expected amplicon sizes are indicated below the gel. (C) Table with primers used.

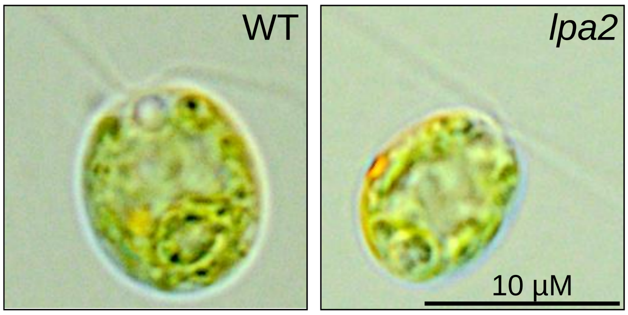


**Supplementary Fig. S2.** Light microscopy images of wild type and *lpa2* mutant cells. Cells were grown in low light (30 µmol photons m$^{-2}$ s$^{-1}$).

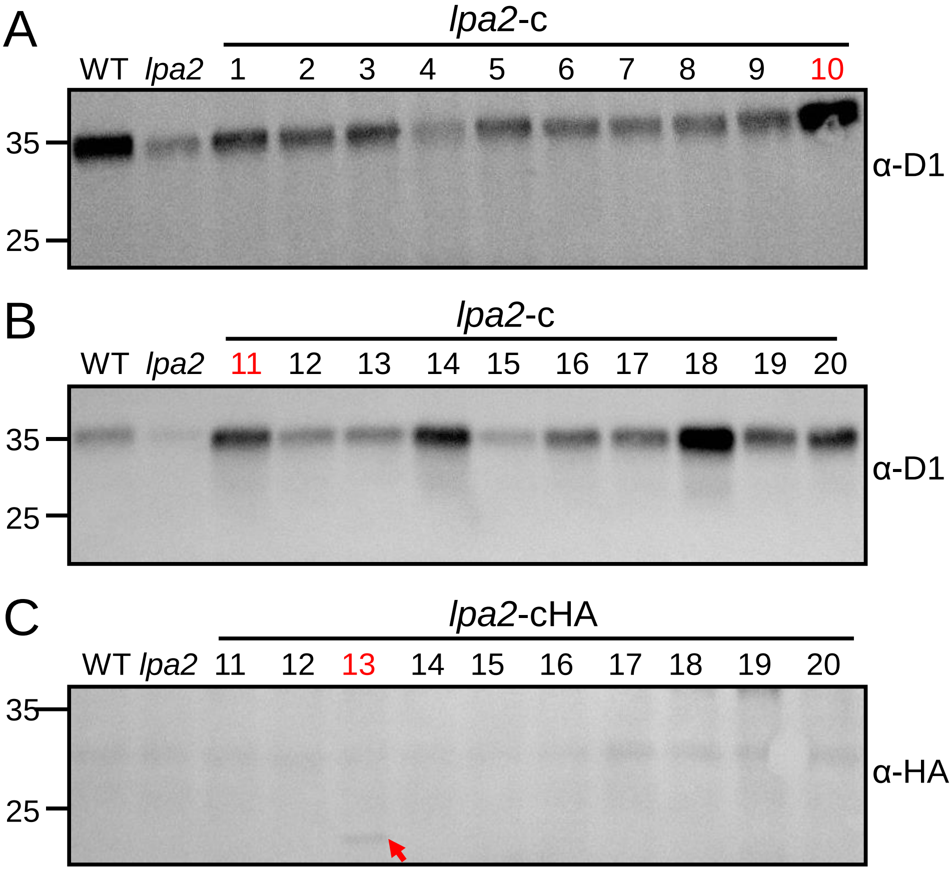


**Supplementary Fig. S3.** Screening of transformants expressing LPA2 with (lpa2-cHA) or without (lpa2-c) a 3xHA tag. 10 µg whole-cell protein of each transformant were separated by SDS-PAGE and analyzed by immunoblotting using antibodies against the D1 protein (A and B) or the HA epitope (C). The transformant number is given on top of the panels. The number in red indicates the transformant chosen for further analysis. The red arrow points to the weak signal obtained for LPA2-3xHA. Note that the signals for WT and *lpa2* mutant in panel (B) are too weak, probably because of a blotting problem at the gel margin.

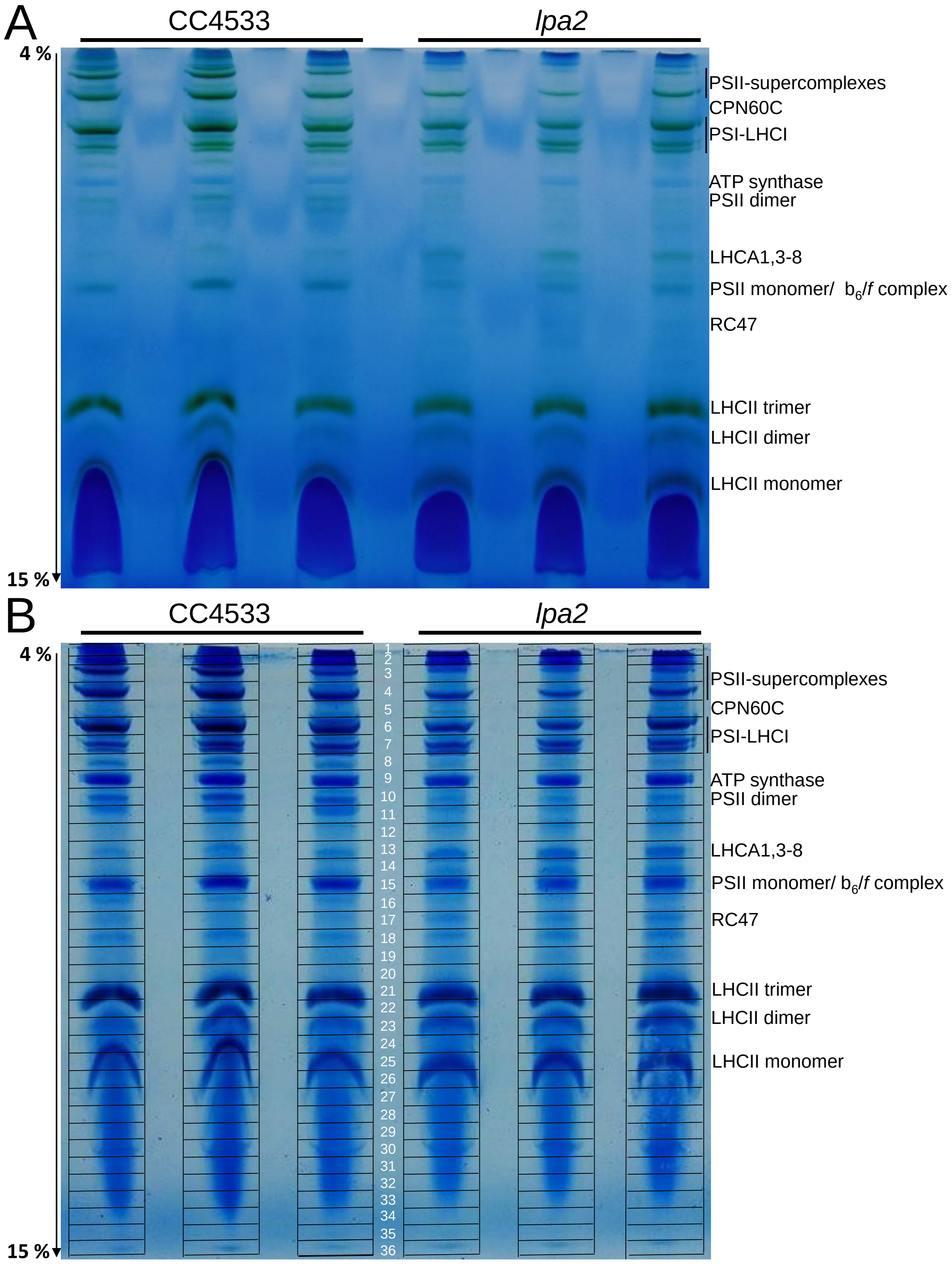


**Supplementary Fig. S4.** BN-PAGE for complexome profiling. Thylakoid membranes were isolated from wild type (CC4533) and *lpa2* mutant cells, solubilized with n-Dodecyl α-D-maltoside, and separated on a 4% to 15% BN gel. 60 µg of protein were loaded per lane. (A) Photography of the gel right after the run. (B) Coomassie staining of the gel shown in (A). Each lane was cut into 36 slices according to the grid shown and gel slices were subjected to tryptic in-gel digestion followed by LC-MS/MS. The identity of the indicated complexes derives from the mass spectrometry analysis (Table S1).

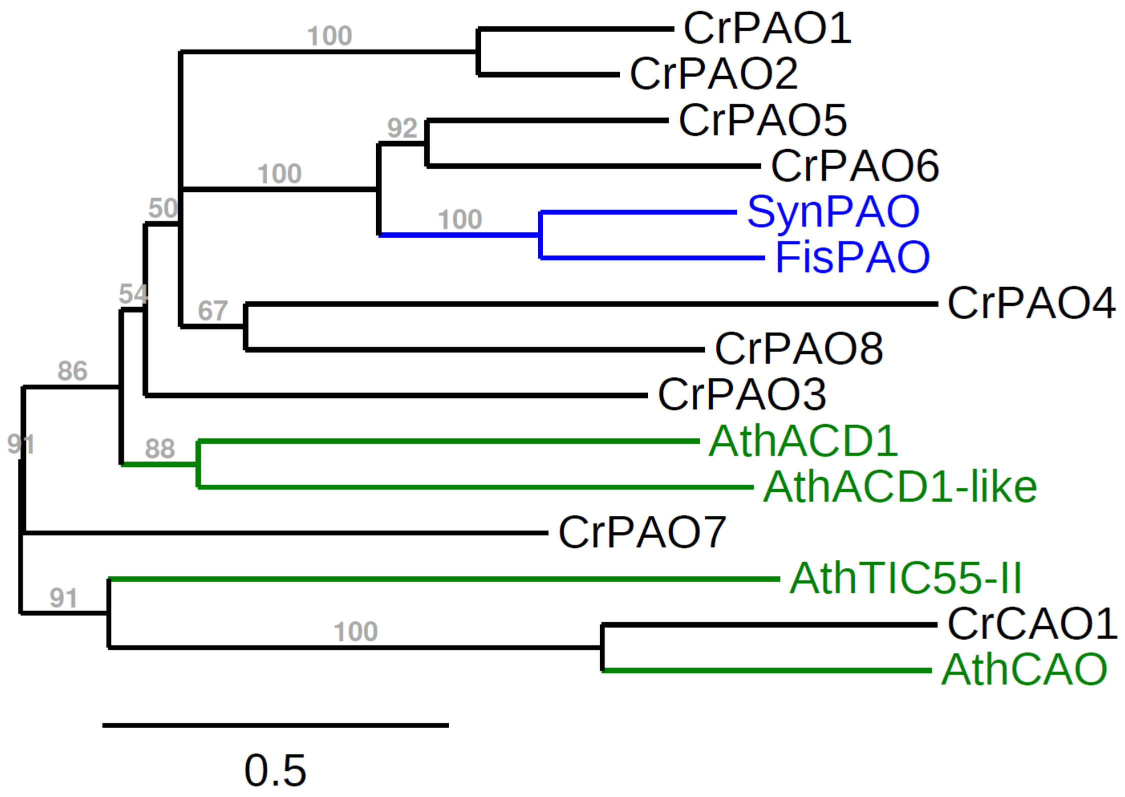


**Supplementary Fig. S5.** Phylogenetic tree of pheophorbide a oxygenases. Sequences are from *Chlamydomonas reinhardtii* (Cr), *Arabidopsis thaliana* (Ath) and the closest homologs to CrPAO5 from *Synechococcus sp.* PCC 7335 (Syn) and *Fischerella sp.* PCC 9431 (Fis). Support for the branches is given in bootstrap values based on 1,000 NJ bootstrap replicates. The sequences are documented in Supplemental Table S2. Accession numbers: CrPAO1: Cre17.g724700; CrPAO2: Cre17.g724600; CrPAO3: Cre10.g450550; CrPAO4: Cre03.g173450; CrPAO5: Cre06.g278245; CrPAO6: Cre06.g305650; CrPAO7: Cre11.g476500; CrPAO8: Cre13.g583050; CrCAO1: Cre01.g043350; SynPAO: EDX87484; FisPAO: WP_155959184; AthACD1: AT3G44880; AthACD1-like: AT4G25650; AthTIC55-II: AT2G24820; AthCAO: AT1G44446.

## SUPPLEMENTARY REFERENCES

**Lu Y**. 2016. Identification and roles of photosystem II assembly, stability, and repair factors in *Arabidopsis*. Front Plant Sci 7, 168.
